# Novel quinazolines bearing 1,3,4-thiadiazole-aryl urea derivative as anticancer agents: design, synthesis, molecular docking, DFT and bioactivity evaluations

**DOI:** 10.1186/s13065-024-01119-0

**Published:** 2024-02-12

**Authors:** Sara Masoudinia, Marjaneh Samadizadeh, Maliheh Safavi, Hamid Reza Bijanzadeh, Alireza Foroumadi

**Affiliations:** 1 Department of Chemistry, Islamic Azad University, Central Tehran Branch, Tehran, Iran; 2 Department of Biotechnology, Iranian Research Organization for Science and Technology (IROST), Tehran, Iran; 3 Department of Environment, Faculty of Natural Resources and Marine Sciences, Tarbiat Modares University, Tehran, Iran; 4 Department of Medicinal Chemistry, Faculty of Pharmacy, Tehran University of Medical Sciences, Tehran, Iran; 5 Drug Design and Development Research Center, The Institute of Pharmaceutical Sciences (TIPS), Tehran University of Medical Sciences, Tehran, Iran

**Keywords:** Quinazoline, Anticancer, Molecular docking, In silico *ADME*, MTT assay

## Abstract

A novel series of 1-(5-((6-nitroquinazoline-4-yl)thio)-1,3,4-thiadiazol-2-yl)-3-phenylurea derivatives **8** were designed and synthesized to evaluate their cytotoxic potencies. The structures of these obtained compounds were thoroughly characterized by IR, ^1^H, and ^13^C NMR, MASS spectroscopy and elemental analysis methods. Additionally, their in vitro anticancer activities were investigated using the MTT assay against A549 (human lung cancer), MDA-MB231 (human triple-negative breast cancer), and MCF7 (human hormone-dependent breast cancer). Etoposide was used as a reference marketed drug for comparison. Among the compounds tested, compounds 8b and 8c demonstrated acceptable antiproliferative activity, particularly against MCF7 cells. Considering the potential VEGFR-2 inhibitor potency of these compounds, a molecular docking study was performed for the most potent compound, **8c**, to determine its probable interactions. Furthermore, computational investigations, including molecular dynamics, frontier molecular orbital analysis, Fukui reactivity descriptor, electrostatic potential surface, and in silico *ADME* evaluation for all compounds were performed to illustrate the structure–activity relationship (SAR).

**Supplementary Information:**

The online version contains supplementary material available at 10.1186/s13065-024-01119-0.

## Introduction

Cancer is a complex and severe human disease characterized by abnormal cell growth that can potentially spread to other parts of the body. It is currently recognized as the second leading cause of death worldwide [1]. Developed countries are experiencing an alarming increase in cancer-related mortality rates, with approximately 10 million deaths recorded in 2020. Predictions suggest that this number will rise to 16.3 million by 2040 [2, 3].

In response to this growing concern, extensive efforts have been made in the field of medicinal chemistry to develop innovative multi-target chemotherapeutics. Various compounds with anticancer properties, such as thiadiazole, quinazolines, and urea derivatives, have been explored [4–12]. Quinazoline, in particular, is a significant aza-heterocycle that not only exhibits strong anti-proliferative effects but also demonstrates a wide range of biological activities, including antioxidant, antibacterial, anti-inflammatory, antimutagenic, antifungal, and antiviral properties [13–21].

Furthermore, several commercially available anticancer drugs, including Erlotinib, Lapatinib, Vandetanib, Afatinib, and Gefitinib, contain quinazoline as a key component and are commonly used worldwide.

Thiadiazole is another common 5-membered heterocyclic system containing one sulfur atom and two nitrogen atoms. This heterocyclic core structure is present in numerous commercially available drugs and biologically active natural products. The remarkable aromaticity, non-toxicity, and exceptional in vivo stability of thiadiazole make it highly desirable for use in bioactive compounds [22].

In recent studies, various thiadiazoles, particularly 1,3,4-thiadiazole derivatives, have been extensively investigated for their potential antitumor activity These compounds have shown significant potency, attracting considerable attention as a promising avenue of research for developing potential anti-cancer agents [23–31].

The urea functionality plays a crucial role in drug development and medicinal chemistry due to its ability to form multiple stable hydrogen bonds with various protein and receptor targets. Urea and its derivatives have been utilized to modulate drug potency, selectivity, and improve drug properties in the development of anticancer, antibacterial, anticonvulsive, anti-HIV, and antidiabetic agents [32–34].

**Fig. 1:**
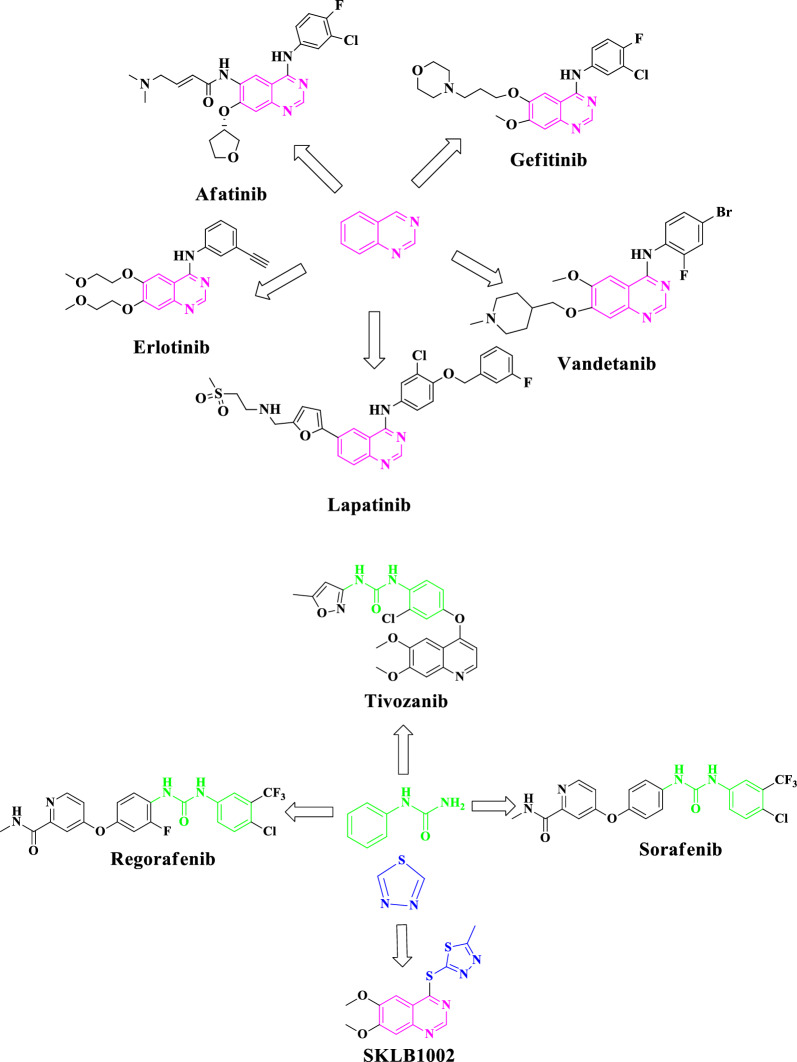
Structures of FDA-approved EGFR and VEGFR-2 inhibitor drugs featuring quinazoline, thiadiazole, and urea moieties

Notably, several FDA-approved drugs containing the urea functionality, such as Regorafenib, Sorafenib, and Tivozanib, have been successfully developed and are widely used as effective anticancer drugs in the global market [35–37] (Fig. 1)

Considerable attention has been focused on ongoing research dedicated to discovering innovative and effective anti-cancer compounds that can target multiple pathways involved in the growth and spread of cancer cells. The process of angiogenesis, which encompasses the formation and maintenance of blood vessels, plays a vital role in cell development and replication. In cancer cells, this physiological process takes on a similar role and becomes crucial in the progression and metastasis of tumors. Therefore, utilizing efficient anti-angiogenesis agents may be proposed as a suitable approach to combat cancer [38].

Vascular endothelial growth factors (VEGF) and their receptors (VEGFR) have been proven to play significant regulatory roles in pathological angiogenesis, particularly in cancer. Among the three subtypes of VEGFRs, VEGFR-2 plays a critical role in promoting tumor angiogenesis. The interaction between VEGF and VEGFR-2 results in dimerization and subsequent auto-phosphorylation of tyrosine residues (Tyr1059 and Tyr1054) within the receptor. This activation initiates signaling pathways that contribute to angiogenesis in the tumor microenvironment [39].

Hence, the use of antitumor substances that disrupt tyrosine phosphorylation demonstrates inhibitory effects on angiogenesis [40]. Therefore, numerous compounds bearing valuable scaffolds particularly quinazolines, thiadiazole, and urea functionality showing great VEGFR-2 inhibitory potencies have been developed. For example, the compound SKLB1002 is a type of quinazoline-bearing 1,3,4-thiadiazole that has been identified as a potent inhibitor of VEGFR-2.

It has shown minimal toxicity in studies [41, 42]. Diaryl ureas have traditionally been used to inhibit VEGFR-2 activity, but there have been advancements in modifying their structure to enhance their effectiveness. For instance, researchers have substituted the urea functional group with a thiourea moiety and incorporated additional rings such as 1,3,4-thiadiazole, oxadiazole, and 1,2,3-triazole to improve binding affinity with the receptor [22, 43, 44].

**Fig. 2:**
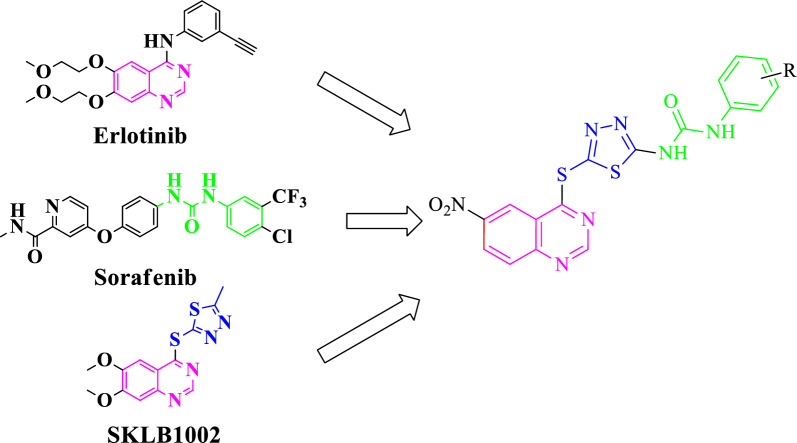
Design strategy of the target 1-(5-((6-nitroquinazolin-4-yl)thio)-1,3,4-thiadiazol-2-yl)-3-phenylurea derivatives as anticancer agents

In the present study, the researchers aimed to develop novel quinazoline conjugates with 1,3,4-thiadiazole and diaryl urea, and to investigate their anticancer activities [45–51]. Therefore, an efficient, multi-step, synthetic pathway was executed to produce the desired compounds, which were then assessed for their ability to inhibit cell proliferation in A549 cells (human lung cancer), MDA-MB-231 (human triple-negative breast cancer), and MCF7 (human hormone-dependent breast cancer) in comparison with Etoposide as the reference drug. Moreover, comprehensive computational investigations, including molecular dynamics, molecular docking studies, frontier molecular orbital analysis using DFT calculations, and in silico* ADME* studies, were performed to confirm the biological results (Fig. 2)

To reveal the significant role of the nitro group on this scaffold, it must be noted this functionality is a unique functional group in medicinal chemistry. It has a powerful electron-attracting potency that creates localized electron-deficient locations within molecules and interacts with biological nucleophiles present in living systems, such as amino acids, proteins, nucleic acids, and enzymes. The interaction can happen by nucleophilic addition and electron transfer involving reduction and oxidation, or also simply by molecular complexation, to induce undesired or desired biological changes [52, 53]. Therefore, countless medicinal chemistry campaigns have been initiated to investigate compounds containing nitro groups. Already, drugs bearing nitro groups have a long history of use as antibiotic, antineoplastic, and antiparasitic agents, as well as fungicides, tranquilizers, insecticides, and herbicides [53–59]. Moreover, some medicinal compounds including PR-104, Tarloxotinib (TH-4000), and Paclitaxel prodrug possess nitro group [60, 61].

## Experimental section

### Materials

The solvents and reagents utilized in this study were obtained from reputable companies such as Merck or Sigma, and no additional purification was required. Melting points were measured using a Kofler hot-plate microscope apparatus, and the values reported were unadjusted. The NMR spectra were obtained using a Bruker FT-500 and Bruker FT-300 MHz spectrometer, with either DMSO-*d6* or CDCl_3_ serving as the solvent, while TMS was used as the internal standard. The chemical shifts and coupling constants are presented in *δ* (ppm) and *J* (Hz), respectively. Additionally, all reactions underwent monitoring via TLC on plastic sheets coated with silica gel 60 F254. The Perkin-Elmer Spectrum Version 10.03.06 was used to obtain IR spectra, specifically using potassium bromide disks. Mass spectra were obtained using the HP Agilent Technologies 5937 instrument with an ionization potential of 70 eV. Elemental analysis for carbon (C), hydrogen (H), and nitrogen (N) was conducted using the elemental analyzer GmbH VarioEL.

### General chemistry

#### Synthesis of quinazoline-4(3*H*)-one (2)

A mixture of 2-aminobenzoic acid **1** (0.137 g, 1 mmol) and formamide (0.045 g, 14 mmol) was heated with vigorous stirring at 150 ℃ for 6h. After completion of the reaction according to the TLC analysis, the reaction mixture was cooled down to the ambient temperature. The precipitated product was filtered and washed with sufficient amount of water to remove the excess of formamide and to afford pure compound as a white solid with m.p. 212–214 ℃ [62].

#### Synthesis of 6-nitroquinazolin-4(3*H*)-one (3)

Under the ice bath conditions, quinazoline-4(3H)-one (0.146 g, 1 mmol) **2** was slowly added into an acid mixture (concentrated sulfuric acid and concentrated nitric acid with a ratio of 1:1) (2 mL) within almost 1h. When the addition was complete, the temperature of the system was slowly raised up to 95 °C, and at this temperature, the reaction was heated for 1 h. After completion of the reaction according to the TLC analysis, the reaction mixture was poured into ice water (25 mL), and it was stirred till yellow solid was precipitated. The precipitation was filtered to afford the pure 6-nitroquinazolin-4(3H)-one **3** with m.p. 279–283 ℃ [63]. ^1^H NMR (500 MHz, DMSO-*d6*) *δ* 11.68 (s, 1H), 8.76 (d, *J* = 2.7 Hz, 1H), 8.57–8.52 (m, 1H), 8.48 (s, 1H), 7.85 (d, *J* = 9.0 Hz, 1H).

#### Synthesis of 4-Chloro-6-nitroquinazoline (4)

6-Nitroquinazolin-4(3H)-one **3** (0.209 g, 1 mmol) was added to the solution of thionyl chloride (SOCl_2_, 2 mL) in DMF (5 mL). The mixture was heated under reflux with stirring for almost 4 h. Then, ice MeOH (15 mL) was added slowly, and the obtained mixture was extracted with dichloromethane (DCM). The organic layer was dried using MgSO_4_ and then concentrated to give 4-chloro-6-nitroquinazoline **4** as a yellow solid with m.p. 134–135 ℃ [64]. ^1^H NMR (500 MHz, DMSO-*d6*): *δ* 8.81 (d, *J* = 2.7 Hz, 1H), 8.56 (dd, *J* = 9.0, 2.7 Hz, 1H), 8.37 (s, 1H), 7.89 (d, *J* = 8.9 Hz, 1H).

#### Synthesis of 5-amino-1,3,4-thiadiazole-2-thiol (6)

In a reaction flask, thiosemicarbazide 5 (1 mmol) and Na_2_CO_3_ (1 mmol) were combined with absolute ethanol (5 mL) and heated to 60 °C for 30 min. A solution of carbon disulfide (CS_2_, 3 mmol) in absolute EtOH (5 mL) was then slowly added drop by drop to the mixture. The resulting mixture was refluxed overnight. Once the reaction was complete, as confirmed by TLC analysis, the solvent was evaporated under reduced pressure. The remaining residue was diluted with water (25 mL), followed by the slow addition of concentrated HCl solution (5 mL). This resulted in the precipitation of compound 6 as a light yellow solid with a yield of 78% and a melting point range of 233–235 [65].

#### Synthesis of 1-(5-mercapto-1,3,4-thiadiazol-2-yl)-3-phenylurea derivatives (8a-l)

5-Amino-1,3,4-thiadiazole-2-thiol (0.133 g, 1 mmol) was dissolved in acetonitrile (CH_3_CN) (3 mL) and stirred at ambient temperature for 30 min. Next, the aryl isocyanate derivative 7a-l (1.2 mmol) was dissolved in CH_3_CN (3 mL) and slowly added to the solution while stirring. The resulting mixture was stirred overnight at room temperature. The solid that formed was filtered and washed with diethyl ether (Et_2_O), resulting in pure white solids known as adducts 8a-l. These adducts were then used in the subsequent step without requiring any additional purification steps [66].

#### Synthesis of 1-(5-((6-nitroquinazolin-4-yl)thio)-1,3,4-thiadiazol-2-yl)-3-phenylurea Derivatives (8a-l)

In order to synthesize the desired products 8a-l, a solution of 1-(5-mercapto-1,3,4-thiadiazol-2-yl)-3-phenylurea derivatives 7a-l (1 mmol) in acetonitrile (5 mL) was prepared. To this solution, a mixture of 4-chloro-6-nitroquinazoline (0.209 g, 1 mmol) and Et_3_N (0.101 g, 1 mmol) was added. The resulting mixture was then refluxed for 3 h. Once the reaction was complete as confirmed by TLC analysis, the solvent was evaporated under reduced pressure and the crude product obtained underwent recrystallization from DMF/Water. This process yielded pure desired products 8a-l in high yields.

##### 1-(5-((6-Nitroquinazolin-4-yl)thio)-1,3,4-thiadiazol-2-yl)-3-phenylurea (8a)

Yellow solid; yield: 68%; mp: 225–227 °C; IR (KBr) (*ν*_max_/cm^–1^): 3247, 1697 (C=O), 1562, 1311. ^1^HNMR (300 MHz, DMSO-*d*_*6*_): *δ* 10.80 (s, 1H), 9.18 (s, 1H), 8.98 (s, 1H), 8.73 (d, *J* = 9.2 Hz, 1H), 8.23 (d, *J* = 9.1 Hz, 1H), 7.74 (d, *J* = 8.1 Hz, 2H), 7.31 (dd, *J* = 7.9 Hz, 2H), 6.99 (dd, *J* = 7.3 Hz, 1H); ^13^C NMR (75 MHz, DMSO-*d*_*6*_): *δ* 170.51, 169.14, 155.81, 154.96, 150.67, 145.60, 145.03, 139.75, 130.94, 128.76 (2CH), 128.22, 122.15, 121.41, 120.22, 118.43 (2CH); ESI-MS m/z: 426.0 [M + H]^+^; Anal. Calcd for C_17_H_11_N_7_O_3_S_2_: C, 47.99; H, 2.61; N, 23.05. Found: C, 48.64; H, 2.55; N, 23.44.

##### 1-(5-((6-Nitroquinazolin-4-yl)thio)-1,3,4-thiadiazol-2-yl)-3-(m-tolyl)urea (8b)

Yellow solid; yield: 78%; mp: 240–24 °C; IR (KBr) (*ν*_max_/cm^–1^): 3370, 1612 (C=O), 1554, 1319; ^1^HNMR (300 MHz, DMSO-*d*_*6*_): *δ* 10.27 (s, 1H), 9.19 (s, 1H), 9.00 (s, 1H), 8.74 (d, *J* = 9.0 Hz, 1H), 8.25 (d, *J* = 8.9 Hz, 1H), 7.56 (s, 1H), 7.44 (d, *J* = 9.3 Hz, 1H), 7.18 (dd, *J* = 7.5 Hz, 1H), 6.81 (d, *J* = 7.2 Hz, 1H), 2.28 (s, 3H); ^13^C NMR (75 MHz, DMSO-*d*_*6*_): *δ* 170.34, 168.64, 155.74, 154.54, 150.64, 145.55, 145.22, 139.49, 137.86, 130.92, 128.55, 128.16, 122.90, 121.36, 120.14, 118.94, 115.58, 21.29; ESI-MS m/z: 440.0 [M + H]^+^; Anal. Calcd for C_18_H_13_N_7_O_3_S_2_: C, 49.19; H, 2.98; N, 22.31. Found: C, 49.43; H, 3.12; N, 23.92.

##### 1-(5-((6-Nitroquinazolin-4-yl)thio)-1,3,4-thiadiazol-2-yl)-3-(p-tolyl)urea (8c)

Yellow solid; yield: 75%; mp: 257–259 °C; IR (KBr) (*ν*_max_/cm^–1^): 3390, 1673 (C=O), 1538, 1322. ^1^HNMR (300 MHz, DMSO-*d*_*6*_): *δ* 10.25 (s, 1H), 9.19 (s, 1H), 9.00 (s, 1H), 8.74 (d, *J* = 9.2 Hz, 1H), 8.25 (d, *J* = 9.2 Hz, 1H), 7.56 (d, *J* = 8.5 Hz, 2H), 7.09 (d, *J* = 8.1 Hz, 2H), 2.24 (s, 3H). ^13^C NMR (75 MHz, DMSO-*d*_*6*_): *δ* 170.45, 169.17, 155.76, 154.86, 150.64, 145.53, 144.89, 137.21, 130.91, 129.12, 128.13 (2CH), 121.36, 120.15, 118.38, 20.40; ESI-MS m/z: 440.0 [M + H]^+^; Anal. Calcd for C_18_H_13_N_7_O_3_S_2_: C, 49.19; H, 2.98; N, 22.3. Found: C, 48.13; H, 3.65; N, 23.42.

##### 1-(3-Methoxyphenyl)-3-(5-((6-nitroquinazolin-4-yl)thio)-1,3,4-thiadiazol-2-yl)urea (8d)

Yellow solid; yield: 62%; mp: 228–230 °C; IR (KBr) (*ν*_max_/cm^–1^): 3386, 1716 (C=O), 1550, 1322. ^1^HNMR (500 MHz, DMSO-*d*_*6*_): *δ* 11.27 (s, 1H), 9.19 (s, 1H), 9.09 (s, 1H), 8.96 (s, 1H), 8.73 (d, *J* = 9.1 Hz, 1H), 8.24 (d, *J* = 9.1 Hz, 1H), 8.24 (dd, *J* = 9.1 Hz, 1H), 7.14 (s, 1H), 6.99 (d, *J* = 8.2 Hz, 1H), 6.64 (d, *J* = 8.4 Hz, 1H), 3.73 (s, 3H), ^13^C NMR (125 MHz, DMSO-*d*_*6*_): *δ* 170.41, 168.49, 155.65, 152.56, 143.29, 142.59, 139.19, 133.09, 130.98, 128.09,127.47, 119.37, 117.23, 112.01, 55.21; ESI-MS m/z: 455.0 [M + H]^+^; Anal. Calcd for C_18_H_13_N_7_O_4_S_2_: C, 47.47; H, 2.88; N, 21.53. Found: C, 48.23; H, 2.31; N, 22.48.

##### 1-(4-Methoxyphenyl)-3-(5-((6-nitroquinazolin-4-yl)thio)-1,3,4-thiadiazol-2-yl)urea (8e)

Yellow solid; yield: 69%; mp: 242–244 °C; IR (KBr) (*ν*_max_/cm^–1^): 3390, 1712 (C=O), 1550, 1326. ^1^HNMR (300 MHz, DMSO-*d*_*6*_): *δ* 11.31 (s, 1H), 9.20 (s, 1H), 8.978 (s, 1H), 8.74 (d, *J* = 6.8 Hz, 1H), 8.25 (d, *J* = 9.2 Hz, 1H), 7.39 (d, *J* = 9.0 Hz, 2H), 6.91 (d, *J* = 9.0 Hz, 2H), 3.73 (s, 3H), ^13^C NMR (75 MHz, DMSO-*d*_*6*_): *δ* 169.82, 164.52, 159.18, 155.73, 155.46, 150.70, 147.57, 130.99, 128.41, 121.44, 120.97 (2CH), 120.27, 114.09 (2CH), 55.20; ESI-MS m/z: 455.0 [M + H]^+^; Anal. Calcd for C_18_H_13_N_7_O_4_S_2_: C, 47.47; H, 2.88; N, 21.53. Found: C, 46.52; H, 2.07; N, 22.98.

##### 1-(3-Fluorophenyl)-3-(5-((6-nitroquinazolin-4-yl)thio)-1,3,4-thiadiazol-2-yl)urea (8f)

Yellow solid; yield: 72%; mp: 230–232 °C; IR (KBr) (*ν*_max_/cm^–1^): 3386, 1720 (C=O), 1592, 1322. ^1^HNMR (500 MHz, DMSO-*d*_*6*_): *δ* 11.45 (s, 1H), 9.47 (s, 1H), 9.20 (s, 1H), 8.95 (s, 1H), 8.73 (d, *J* = 9.1 Hz, 1H), 8.25 (d, *J* = 9.1 Hz, 1H), 7.46 (d, *J* = 11.5 Hz, 1H), 7.35 (d, *J* = 6.8 Hz, 1H), 7.22 (d, *J* = 8.7 Hz, 1H), 6.88 (dd, *J* = 8.5 Hz, 1H). ^13^C NMR (125 MHz, DMSO-*d*_*6*_): *δ* 170.15, 168.02, 159.72, 156.67, 152.40, 147.57, 145.64, 143.11, 133.12, 131.07, 130.91, 128.30, 124.02, 122.99, 121.40, 119.37, 118.62; ESI–MS m/z: 443.0 [M + H]^+^; Anal. Calcd for C_17_H_10_FN_7_O_3_S_2_: C, 46.05; H, 2.27; N, 22.11. Found: C, 47.35; H, 2.84; N, 23.59.

##### 1-(3-Chlorophenyl)-3-(5-((6-nitroquinazolin-4-yl)thio)-1,3,4-thiadiazol-2-yl)urea (8g)

Yellow solid; yield: 65%; mp: 258–260 °C; IR (KBr) (*ν*_max_/cm^–1^): 3370, 1720 (C=O), 1535, 1322. ^1^HNMR (300 MHz, DMSO-*d*_*6*_): *δ* 10.97 (s, 1H), 9.18 (s, 1H), 8.96 (s, 1H), 8.71 (d, *J* = 9.2 Hz, 1H), 8.23 (d, *J* = 9.2 Hz, 1H), 7.88 (s, 1H), 7.53 (d, *J* = 8.2 Hz, 1H), 7.32 (dd, *J* = 8.1 Hz, 1H), 7.02 (d, *J* = 5.7 Hz, 1H), ^13^C NMR (75 MHz, DMSO-*d*_*6*_): *δ* 170.15, 167.30, 155.77, 154.00, 150.66, 145.65, 140.87, 133.25, 130.96, 130.45, 128.30, 121.99, 120.22, 117.68, 116.78; ESI-MS m/z: 460.0 [M + H]^+^; Anal. Calcd for C_17_H_10_ClN_7_O_3_S_2_: C, 44.40; H, 2.19; N, 21.32. Found: C, 45.35; H, 2.62; N, 22.34.

##### 1-(4-Chlorophenyl)-3-(5-((6-nitroquinazolin-4-yl)thio)-1,3,4-thiadiazol-2-yl)urea (8h)

Yellow solid; yield: 67%; mp: 246–248 °C; IR (KBr) (*ν*_max_/cm^–1^): 3390, 1720 (C=O), 1538, 1322. ^1^HNMR (300 MHz, DMSO-*d*_*6*_): *δ* 11.44 (s, 1H), 9.32 (s, 1H), 9.19 (s, 1H), 8.96 (s, 1H), 8.73 (d, *J* = 9.2 Hz, 1H), 8.24 (d, *J* = 9.2 Hz, 1H), 7.52 (d, *J* = 8.9 Hz, 2H), 7.36 (d, *J* = 8.9 Hz, 2H), ^13^C NMR (75 MHz, DMSO-*d*_*6*_): *δ* 169.61, 160.56, 155.68, 150.69, 145.74, 137.16, 130.99, 128.79 (2CH), 128.41, 126.91, 121.42, 120.61, 120.25 (2CH); ESI-MS m/z: 460.0 [M + H]^+^; Anal. Calcd for C_17_H_10_ClN_7_O_3_S_2_: C, 44.40; H, 2.19; N, 21.32. Found: C, 43.24; H, 2.87; N, 22.43.

##### 1-(4-Bromophenyl)-3-(5-((6-nitroquinazolin-4-yl)thio)-1,3,4-thiadiazol-2-yl)urea(8i)

Yellow solid; yield: 60%; mp: 236–238 °C; IR (KBr) (*ν*_max_/cm^–1^): 3386, 1716 (C=O), 1531, 1319. ^1^HNMR (300 MHz, DMSO-*d*_*6*_): *δ* 10.64 (s, 1H), 9.19 (s, 1H), 9.00 (s, 1H), 8.74 (d, *J* = 6.8 Hz, 1H), 8.25 (d, *J* = 9.2 Hz, 1H), 7.65 (d, *J* = 8.9 Hz, 2H), 7.46 (d, *J* = 8.9 Hz, 2H), ^13^C NMR (75 MHz, DMSO-*d*_*6*_): *δ* 170.45, 159.72, 156.67, 155.84, 150.68, 145.70, 138.94, 131.54 (2CH), 130.95, 128.32, 124.27, 121.45, 120.27 (2CH); ESI-MS m/z: 505.9 [M + H]^+^; Anal. Calcd for C_17_H_10_BrN_7_O_3_S_2_: C, 40.48; H, 2.00; N, 19.44. Found: C, 41.18; H, 2.30; N, 18.14.

##### 1-(3,4-Dichlorophenyl)-3-(5-((6-nitroquinazolin-4-yl)thio)-1,3,4-thiadiazol-2-yl)urea (8j)

Yellow solid; yield: 65%; mp: 252–254 °C; IR (KBr) (*ν*_max_/cm^–1^): 3382, 1727 (C=O), 1585, 1322. ^1^HNMR (300 MHz, DMSO-*d*_*6*_): *δ* 11.54 (s, 1H), 9.44 (s, 1H), 9.19 (s, 1H), 8.91 (d, *J* = 2.5 Hz, 1H), 8.71 (d, *J* = 9.2 Hz, 1H), 8.22 (d, *J* = 9.2 Hz, 1H), 7.83 (s, 1H), 7.52 (d, *J* = 8.8 Hz, 1H), 7.39 (d, *J* = 8.9 Hz, 1H), ^13^C NMR (75 MHz, DMSO-*d*_*6*_): *δ* 170.41, 168.49, 156.69, 155.83, 152.56, 143.29, 139.19, 133.09, 130.98, 128.09, 127.47, 119.37, 118.62, 118.00, 117.23; ESI-MS m/z: 493.9 [M + H]^+^; Anal. Calcd for C_17_H_9_C_l2_N_7_O_3_S_2_: C, 41.30; H, 1.84; N, 19.83. Found: C, 40.35; H, 2.01; N, 20.73.

##### 1-(3-Chloro-4-methylphenyl)-3-(5-((6-nitroquinazolin-4-yl)thio)-1,3,4-thiadiazol-2-yl)urea (8k)

Pale-yellow solid; yield: 69%; mp: 234–236 °C; IR (KBr) (*ν*_max_/cm^–1^): 3178, 1616 (C=O), 1542, 1311. ^1^HNMR (300 MHz, DMSO-*d*_*6*_): *δ* 10.49 (s, 1H), 9.19 (s, 1H), 8.98 (s, 1H), 8.74 (d, *J* = 6.8 Hz, 1H), 8.25 (d, *J* = 9.2 Hz, 1H), 7.84 (s, 1H), 7.43 (d, *J* = 6.0 Hz, 1H), 7.25 (d, *J* = 8.8 Hz, 1H), 2.25 (s, 3H), ^13^C NMR (75 MHz, DMSO-*d*_*6*_): *δ* 170.02, 168.04, 155.64, 154.33, 150.60, 145.64, 145.48, 138.72, 133.12, 131.07, 130.91, 128.49, 128.09, 121.27, 120.04, 118.07, 116.94, 18.83; ESI-MS m/z: 474.0 [M + H]^+^; Anal. Calcd for C_18_H_12_ClN_7_O_3_S_2_: C, 45.62; H, 2.55; N, 20.69. Found: C, 46.70; H, 3.01; N, 21.99.

##### 1-(4-chloro-3-(trifluoromethyl)phenyl)-3-(5-((6-nitroquinazolin-4-yl)thio)-1,3,4-thiadiazol-2-yl)urea (8l)

Yellow solid; yield: 58%; mp: 240–245 °C; IR (KBr) (*ν*_max_/cm^–1^): 3363, 1704 (C=O), 1527, 1319. ^1^HNMR (300 MHz, DMSO-*d*_*6*_): *δ* 11.65 (s, 1H), 9.61 (s, 1H), 9.20 (s, 1H), 8.94 (s, 1H), 8.72 (d, *J* = 9.3 Hz, 1H), 8.24 (d, *J* = 9.1 Hz, 1H), 8.07 (s, 1H), 7.73 (d, *J* = 8.8 Hz, 1H), 7.64 (d, *J* = 8.8 Hz, 1H), ^13^C NMR (75 MHz, DMSO-*d*_*6*_): *δ* 170.15, 163.09, 159.51, 155.77, 154.00, 150.66, 143.11, 133.25, 130.96, 128.49, 128.09, 127.62, 126.91, 121.40, 120.23, 117.67; ESI-MS m/z: 527.9 [M + H]^+^; Anal. Calcd for C_18_H_9_ClF_3_N_7_O_3_S_2_: C, 40.95; H, 1.72; N, 18.57. Found: C, 41.25; H, 1.89; N, 19.53.

### In vitro cytotoxicity assay

The MTT assay was used to test the cytotoxicity of novel 6-nitroquinazoline conjugated with 1,3,4-thiadiazole and diaryl-urea **8** against the A-549 (human lung cancer), MCF-7 (human hormone dependent breast cancer), MDA-MB-231 (human triple negative breast cancer), and HDF (Human Dermal Fibroblasts) cell lines. All of the cell lines were obtained from the Pastor Institute's National Cell Bank in Tehran, Iran, and were cultured in either RPMI-1640 or DMEM supplemented with 10% fetal bovine serum (Gibco, Milano, Italy). The cancerous cells were cultured at 37 °C in a humidified 5% CO_2_ incubator after being seeded onto 96-well micro-plates at a density of 1 × 10^4^ cells/mL. After overnight incubation at 37 °C, compounds were added to the cells at varying final concentrations (1 µg/mL, 5 µg/mL, 20 µg/mL, and 40 µg/mL) after being dissolved in dimethyl sulfoxide (DMSO). Fresh phenol red free RPMI-1640 containing 0.5 mg/mL of MTT was added after incubation. Using a multi-well plate reader (Gen5, Epoch, BioTek) optical density was measured at 492 nm. Both Etoposide and 1% DMSO were employed as positive and negative controls, respectively. Three separate dose–response curves were used to get the IC_50_ values (the dosage needed to suppress cell growth by 50%).

## Results and discussion

### Chemistry

**Scheme 1:**
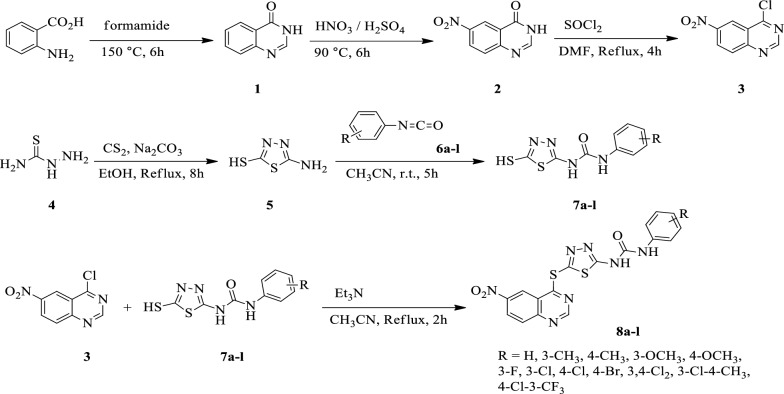
Synthetic route toward 6-nitroquinazoline conjugated with 1,3,4-thiadiazole and diaryl-urea **8a-l**

The synthetic approach toward the desired novel 6-nitroquinazoline conjugated with 1,3,4-thiadiazole and diaryl-urea **8a-l** is illustrated in Scheme 1. Heating a mixture of 2-aminobenzoic acid and formamidine under the reflux conditions led to obtain quinazoline-4(3*H*)-one **1** which subsequently underwent the electrophilic substitution with the mixture of nitric acid and sulfuric acid at the ambient temperature to give 6-nitroquinazolin-4(3*H*)-one **2** The chlorination of carbonyl group in this compound with thionyl chloride in DMF was carried out the reflux conditions to obtain 4-chloro-6-nitroquinazolin-4(3*H*)-one **3**. On the other hand, the production of 1-(5-mercapto-1,3,4-thiadiazol-2-yl)-3-arylurea 7a-l was achieved through a condensation reaction involving thiosemicarbazide 4 and carbon disulfide. This reaction took place in the presence of sodium carbonate under reflux conditions in absolute EtOH (compound 5). Subsequently, various substituted phenyl isocyanates 6a-l were added to the mixture in CH_3_CN to yield the corresponding derivatives 7a-l. Finally, chlorine moiety compound **3** underwent nucleophilic aromatic substitution with synthesized 1-(5-mercapto-1,3,4-thiadiazol-2-yl)-3-arylurea **7a-l** in the presence of Et_3_N in CH_3_CN under the reflux conditions to afford our desirable 6-nitroquinazoline conjugated with 1,3,4-thiadiazole and diaryl-urea derivatives **8a-l**. The spectra of the compounds are provided in the supplementary information file.

The generality of the substrate scope was investigated using various substituted phenyl isocyanates **6a-l** bearing electron-donating groups like methyl and methoxy, as well as electron-withdrawing groups like fluorine, chlorine, bromine, and trifluoromethyl. The structures of isolated 6-nitro quinazoline conjugated with 1,3,4-thiadiazole and diaryl-urea derivatives **8a-l** are summarized in Table 1. They are deduced based on their IR, ^1^H, and ^13^C NMR, as well as MASS spectroscopy and elemental analysis. Partial assignments of these resonances are given in the Experimental Part.

**Table 1:**
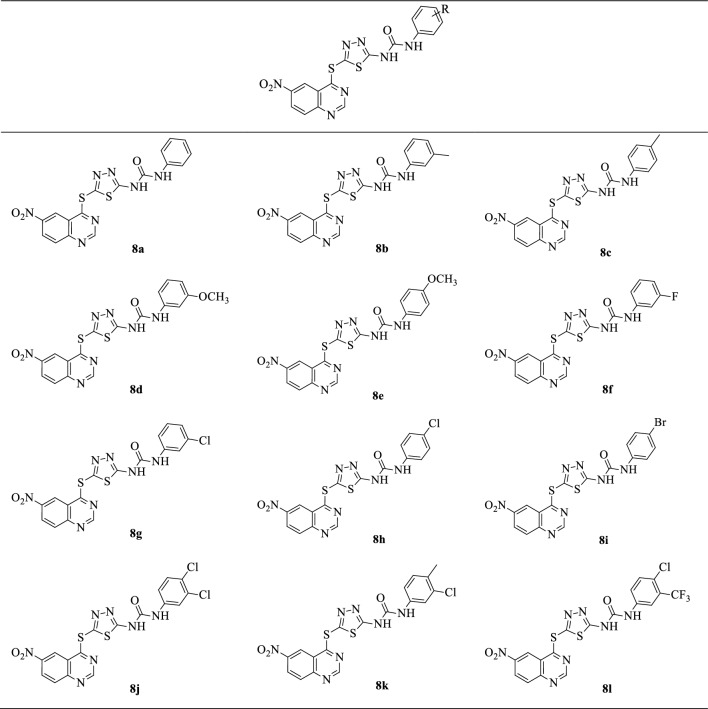
Substrate scope for the synthesis of 6-nitroquinazoline conjugated with 1,3,4-thiadiazole and diaryl-urea **8a-l**

### In Vitro antiproliferative activity

The targeted 6-nitroquinazoline conjugated with 1,3,4-thiadiazole and diaryl-urea **8a-l** were evaluated for their in vitro antitumor activities against three human cancerous cell lines, including A549, MDA-MB231, and MCF7 cell lines by using the MTT colorimetric assay to investigate the role of substituents on the phenyl moiety originated from arylisocyanates. The cytotoxicity is expressed as the concentration which inhibits 50% of cell viability (IC_50_). In present study, Etoposide was used as a positive control having IC_50_ values of 25.8 ± 0.0008 µM against A549, 27.9 ± 0.002 µM against MDA-MB231, and 29.8 ± 0.0007 µM against MCF7. These compounds were shown better cytotoxicity comparable to Etoposide on MCF7 cells, as some derivatives named **8b**, **8c**, and **8g** exhibited IC_50_ values of 29.0 ± 0.005 µM, 25.0 ± 0.001 µM, 39.1 ± 0.001 µM, respectively. Other results were noticeably weaker than standard drug. It seems that the presence of methyl as a mild electron-donating group particularly at C-4 position (compound **8c**) improved the cytotoxic potency (Table 2). Furthermore, we assessed the cytotoxic impact of 8b and 8c as most potent cytotoxic compounds on normal HDF. Remarkably, no toxic properties were observed in HDF cells even at a concentration as high as 50 µM.

**Table 2:** In vitro anti-proliferative effects (IC_50_, µM) of compounds **8a-l** against A549, MDA-MB231 and MCF7 cell lines

Compound	R	A549	MDA-MB231	MCF7
**8a**	H	> 50	> 50	> 50
**8b**	3-CH_3_	> 50	> 50	**29 ± 0.005**
**8c**	4-CH_3_	> 50	> 50	**25 ± 0.001**
**8d**	3-OCH_3_	> 50	> 50	> 50
**8e**	4-OCH3	> 50	> 50	> 50
**8f**	3-F	> 50	> 50	> 50
**8g**	3-Cl	> 50	> 50	**39.1 ± 0.001**
**8h**	4-Cl	> 50	> 50	> 50
**8i**	4-Br	> 50	> 50	> 50
**8j**	3,4DiCl	> 50	> 50	> 50
**8k**	3-Cl-4-CH_3_	> 50	**46 ± 0.003**	> 50
**8l**	4-Cl-3-CF_3_	> 50	> 50	> 50
**Etoposide**	–	**25.8 ± 0.0008**	**27.9 ± 0.002**	**29.8 ± 0.0007**

Values were the means of three replicates ± standard deviation (SD)

### Computational details

To investigate the electronic features of synthesized 6-nitroquinazoline conjugated with 1,3,4-thiadiazole and diaryl-urea **8a-l**, quantum calculations were performed at the B3LYP/6-31 g (d, p) level of theory. Frequency calculations were performed to confirm the nature of minima structures. All the DFT quantum calculations were conducted using GAMESS [67]. The frontier molecular orbital analysis was performed to study the chemical reactivity of ligands **8**. The electrostatic surface potential (ESP) analysis was performed by Multiwfn 3.3.8 code [68]. To reveal the relationship between the structure and activity of compounds, the quantitative ESP analysis was done. To evaluate the plausible interactions between most potent compounds, **8c**, as a potential VEGFR-2 inhibitor, and VEGFR-2 target, the molecular docking calculation were performed using Autodock 4.2.1. The crystallographic structure of 3WZE was selected as a VEGFR-2 target.

Molecular dynamic simulation was performed with GROMACS 2020. Amber force field was used for building the topological files for protein and ligand. To consider the solvation, the TIP3 water model was employed. The box system including protein, ligand, water, and ions was minimized foe 2175 steps. The equilibration process was done in the NVT and NPT ensembles at the pressure of 1 atm and temperature of 300K for 200 ps. The MD simulation was performed for 100 ns. The RMSD, RMSF, and hydrogen bond analysis were performed on ythe trajectory file.

#### Frontier molecular orbital analysis

**Fig. 3:**
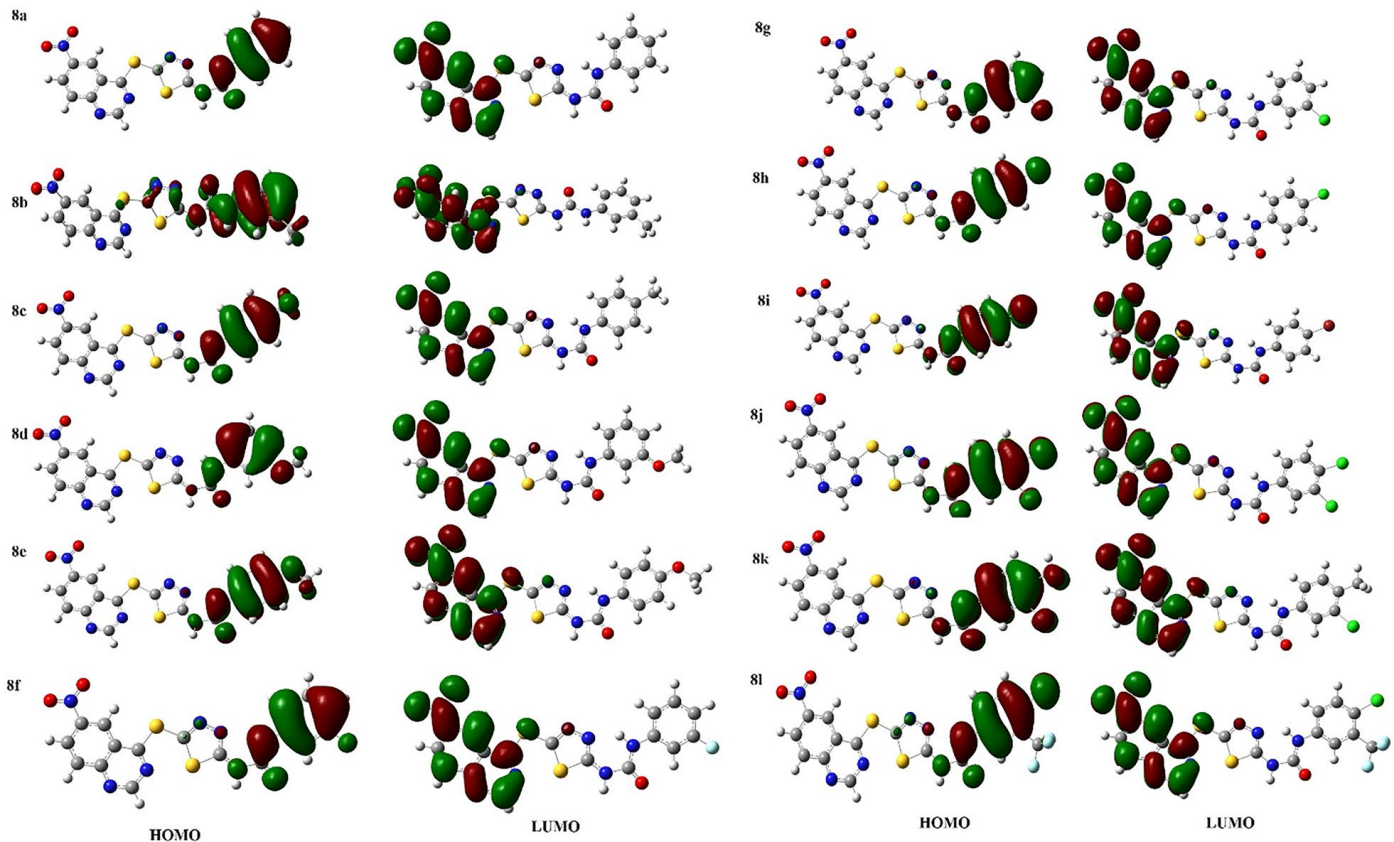
Frontier molecular orbitals of studied ligands

The frontier molecular orbitals could be used to elucidate the chemical reactivity of a molecule. The energy gap can be considered as an indicator of the stability and chemical reactivity. Molecules with smaller band gap categorized as more polarizable molecules with higher chemical reactivity. In all 6-nitroquinazoline conjugated with 1,3,4-thiadiazole and diaryl-urea **8a-l**, HOMO is located on the phenyl urea moieties whereas the LUMO is localized on the nitro quinazoline moiety. Therefore, according to different distribution of HOMO and LUMO, one can expect that there is a favorable intramolecular charge transfer for these series of ligands (Fig. 3).

The HOMO and LUMO orbital energies can be used to assess the quantum chemical reactivity indexes including electrophilicity and nucleophilicity indexes, chemical potential, softness and hardness parameters as:

$$\upmu=\frac{{\text{E}}_{\text{HOMO}}+{\text{E}}_{\text{LUMO}}}{2}\quad\upeta=\frac{{\text{E}}_{\text{LUMO}}-{\text{E}}_{\text{HOMO}}}{2}\quad\upomega=\frac{{\upmu}^{2}}{2\upeta}$$

The quantum chemical indexes including chemical potential, chemical hardness, and global electrophilicity indexes are calculated for all obtained compounds **8a-l**, and the results are summarized in Table 3. These parameters are calculated based on the ground state optimized geometries. It can be seen that the chemical reactivity of all ligands is negative which can be assign to the stability of these compounds **8**.

**Table 3:** The quantum reactivity indexes including chemical potential ($\mu$), hardness ($\eta$), and electrophilicity index ($\omega$) in eV

Compound	${\text{E}}_{{\varvec{g}}}$	$\upmu$	$\upeta$	$\omega$
**8a**	2.68	− 4.59	1.34	7.86
**8b**	2.96	− 4.57	1.48	7.05
**8c**	2.50	− 4.49	1.25	8.06
**8d**	2.49	− 4.47	1.24	8.05
**8e**	2.16	− 4.31	1.08	8.60
**8f**	2.81	− 4.69	1.40	7.85
**8g**	2.88	− 4.74	1.44	7.80
**8h**	2.71	− 4.65	1.35	8.00
**8i**	2.67	− 4.63	1.33	8.06
**8j**	2.86	− 4.77	1.43	7.95
**8k**	2.70	− 4.63	1.35	7.93
**8l**	2.97	− 4.83	1.48	7.88

#### Fukuie reactivity descriptor

A key parameter to describe the reactive area in a molecule is Fukui function, f(r), which is based on the conceptual density functional theory. It can be described as $\left(r\right)={\left[\frac{\partial \rho (r)}{\partial N}\right]}_{\upsilon }$ where N and $\upsilon$ can be defined as number of electrons in the system and the external potential due to nuclear charges, respectively. Finding the reactive centers is important to elucidate the interaction between the compound and biological target. For this purpose, the Fukui parameters are calculated for the most potent 6-nitroquinazoline conjugated with 1,3,4-thiadiazole and diaryl-urea, **8c**. In general, the centers with high activity show the more values of Fukui functions. This function can be evaluated for three conditions including nucleophilic attack ${f}^{+}(r)$, electrophilic attack ${f}^{-}(r)$, and radical attack ${f}^{0}(r)$ according to:

$${f}^{+}(r)={\rho }_{N+1}\left(r\right)-{\rho }_{N}(r)$$

$${f}^{-}(r)={\rho }_{N}\left(r\right)-{\rho }_{N-1}(r)$$

$${f}^{0}(r)=\frac{{\rho }_{N+1}\left(r\right)-{\rho }_{N-1}(r)}{2}$$

Dual descriptor is another parameter for the evaluation of the nucleophilic and electrophilic centers at the same time. The regions with $\Delta f>0$ and $\Delta f<0$ can be described as suitable centers for a nucleophilic attack and electrophilic attack, respectively. $\Delta f$ can be evaluated as

$$\Delta f\left(r\right)= {f}^{+}\left(r\right)-{f}^{-}\left(r\right)={\rho }_{N+1}\left(r\right)-{2\rho }_{N}\left(r\right)+ {\rho }_{N-1}(r)$$

**Fig. 4:**
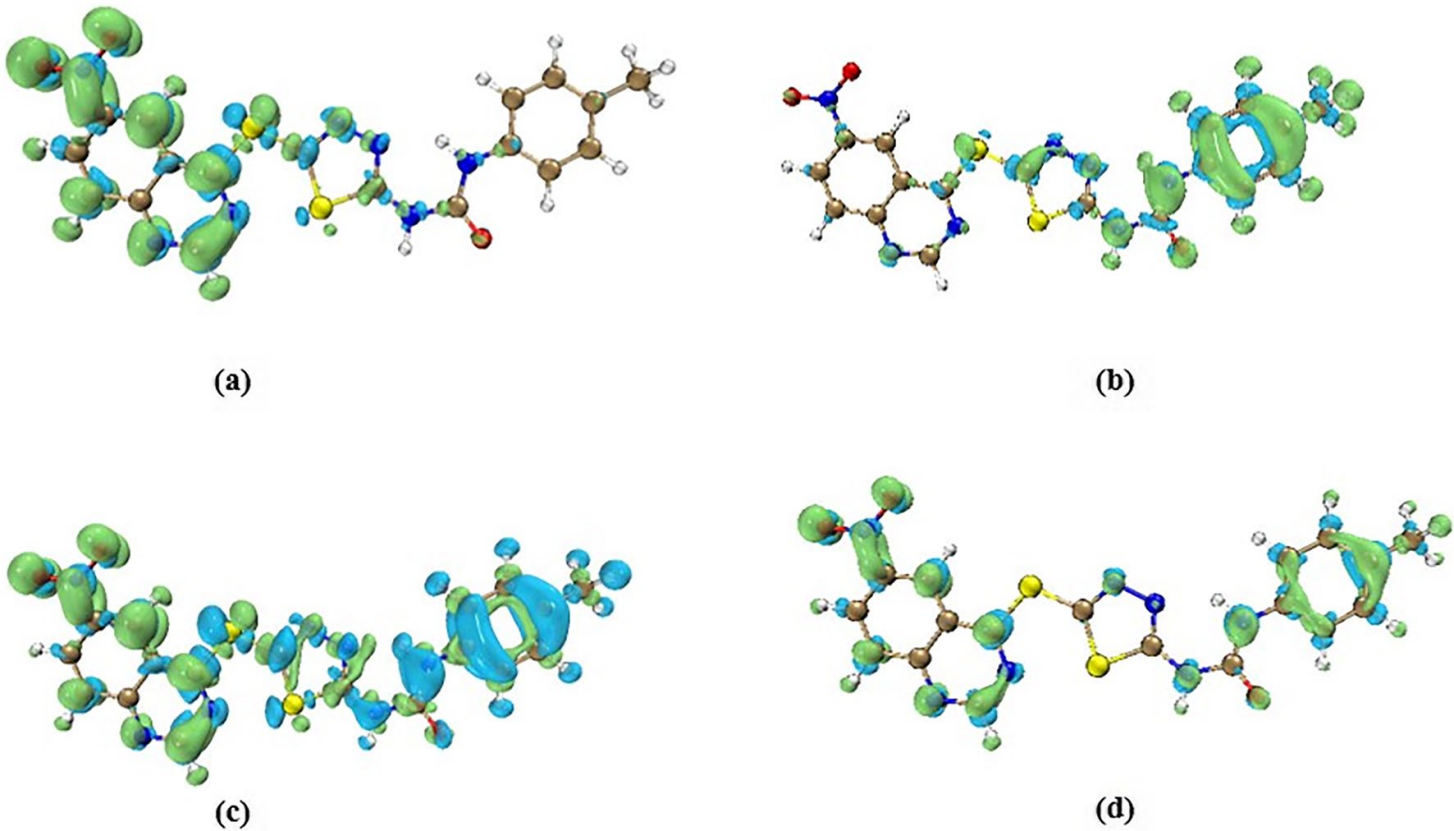
Fukui functions for **a** nucleophilic attack *f*^+^(*r*) **b** electrophilic attack f^−^ (r) **c** dual descriptor (Δ*f*), and **d** radical attack f^0^ for **8c** ligand at B3LYP/6–31 g (d, p) level. The green and blue regions show positive and negative values of these functions

The graphical view of these indexes was indicated in Fig. 4.

Furthermore, to quantify the amount of reactivity in each site of the molecules, the condensed Fukui functions are calculated and the results are reported in Table 4.

**Table 4:** The values of Fukui functions *f*^−^(*r*), *f*^+^(*r*), and *f*^0^ (*r*)) and dual descriptors (∆*f*(*r*)) for active sites on the potent **8c** ligand

Atom	q(N)	q(N + 1)	q(N-1)	f^−^	f^+^	f^0^	DD
N39	0.2497	0.1986	0.2524	0.0028	0.0510	0.0269	0.0483
O40	− 0.1935	− 0.2946	− 0.1769	0.0166	0.1011	0.0588	0.0844
O41	− 0.1941	− 0.2842	− 0.1864	0.0077	0.0901	0.0489	0.0824
N42	− 0.1487	− 0.1849	− 0.1450	0.0036	0.0362	0.0199	0.0326
N43	− 0.1598	− 0.2139	− 0.1365	0.0233	0.0541	0.0387	0.0308
C23	0.0130	0.0140	0.0360	0.0230	− 0.0009	0.0110	− 0.0239
C21	0.0820	0.0612	0.0868	0.0048	0.0209	0.0128	0.0160
N4	− 0.0861	− 0.0946	− 0.0528	0.0333	0.0085	0.0209	− 0.0248
C1	0.2080	0.2045	0.2268	0.0188	0.0035	0.0111	− 0.0153
O20	− 0.2963	− 0.3128	− 0.2550	0.0413	0.0165	0.0289	− 0.0249
N2	− 0.0819	− 0.0842	− 0.0224	0.0596	0.0023	0.0309	− 0.0573
C11	− 0.0462	− 0.0502	− 0.0046	− 0.0415	0.0040	0.0228	− 0.0375
C6	0.0447	0.0487	0.1009	0.0562	− 0.0040	0.0261	− 0.0602
C7	− 0.0544	− 0.0572	− 0.0067	0.0477	0.0028	0.0252	− 0.0449
C8	− 0.0568	− 0.0563	− 0.0098	0.0470	− 0.0005	0.0232	− 0.0475
C9	− 0.0437	− 0.0495	− 0.0019	0.0419	0.0058	0.0238	− 0.0361
C13	− 0.0024	− 0.0101	0.0721	0.0745	0.0077	0.0411	− 0.0668
C16	− 0.0817	− 0.0845	− 0.0608	0.0209	0.0027	0.0118	− 0.0181
C28	− 0.0131	− 0.0282	− 0.0091	0.0040	0.0151	0.0096	0.0111
C29	− 0.0202	− 0.0923	− 0.0202	0.0001	0.0721	0.0361	0.0720
C30	0.0654	0.0476	0.0718	0.0065	0.0178	0.0121	0.0113
C31	0.0960	0.0314	0.1019	0.0058	0.0647	0.0352	0.0588
C32	0.0334	− 0.0084	0.0451	0.0117	0.0417	0.0267	0.0300
C34	− 0.0211	− 0.0748	− 0.0098	0.0113	0.0537	0.0325	0.0424
C36	− 0.0159	− 0.0420	− 0.0033	0.0126	0.0261	0.0193	0.0134
S22	0.0762	0.0596	0.1224	0.0462	0.0166	0.0314	− 0.0296
S26	0.0933	0.0477	0.1498	0.0565	0.0456	0.0511	− 0.0109
N24	− 0.1283	− 0.1555	− 0.1053	0.0230	0.0272	0.0251	0.0042
N25	− 0.1300	− 0.1474	− 0.1137	0.0163	0.0174	0.0169	0.0011

It can be seen that regarding the nucleophilic attack, the potential active sites are distributed mainly on the nitro quinazoline part. As to electrophilic attack, the plausible active sites are localized on the phenyl urea moiety. It can be seen that the thiadiazole ring is common in both nucleophilic and electrophilic attacks. To clarify and asses the nature of this position, the dual descriptor parameter could be useful. It can be seen that the DD parameter at thiadiazole ring is mainly positive. Therefore, it has potential for nucleophilic attack. For the other fragments in the compound **8c**, the results of dual parameter are in the line with the results of f^+^(r) and f^−^(r) Fukui functions. For radical attack, the plausible active sites are located mainly on the nitro and phenyl ring.

#### Electrostatic potential surface

**Fig. 5:**
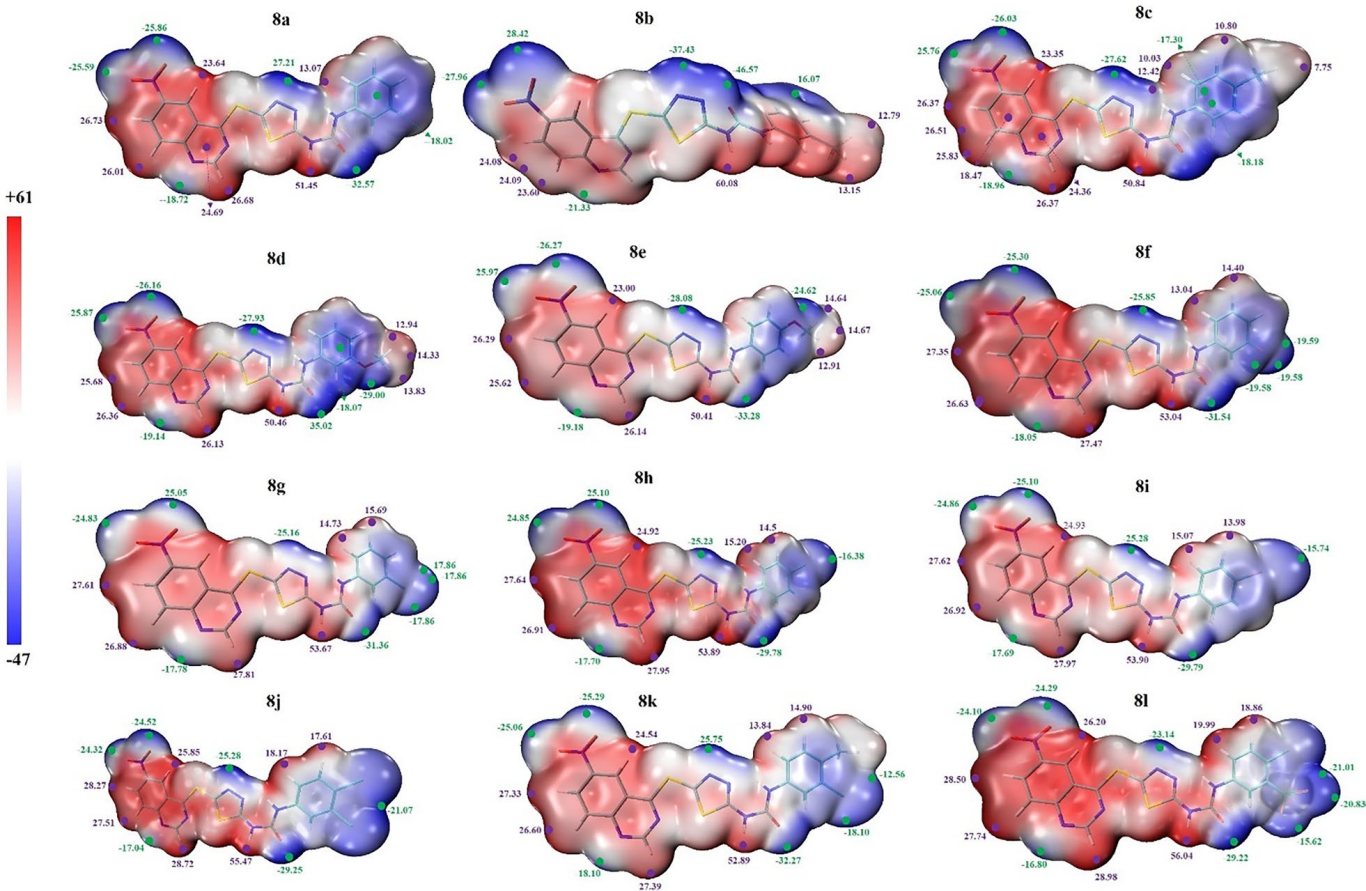
The quantitative ESP analysis on the molecular vdW surface of studied ligands. The unit is in kcal/mol. The green and magenta spheres represent surface local minima and maxima of ESP values, respectively

The electrostatic potential iso-surfaces can be considered as a valuable tool to conduct a close link between the distribution of electrons in a molecule and the biological activity. It can be used to predict and interpret the weak non-covalent interactions between the small ligands and the macromolecule target. The ESP analysis elucidate for us the regions with positive and negative electrostatic potential with low and high amount of electron densities, respectively. The map of electron density for two compounds is plotted in Fig. 5.

The regions with low and high electron densities are shown with red and blue color, respectively. The quantitative electrostatic potential analysis was performed to shed light more accurately on the regions with high and low electron densities based on the optimized structures of molecules on the ground state. The positive regions on the hydrogen atoms are due to the lower electronegativities of this element. The electronegative elements with high electronegativities show negative regions. The white color shows region with moderate electronegativities. It is expected that the regions with negative potential conduct strong attraction with positive regions on the target and the positive regions on the ligand conduct attraction interactions with negative regions on the target. It can be seen that different position of methyl group on compounds **8b** and **8c** led to different distribution of electron density that has an important role in the bioactivity of these compounds.

To have a favorable interaction between ligand and bio target, a complementary electron density distribution must be existed. Different substituents on the phenyl ring led to different distribution of electron density and consequently the different bioactivity. In all 6-nitroquinazoline conjugated with 1,3,4-thiadiazole and diaryl-urea **8a-l**, hydrogen atoms with lower electronegativity show positive potential region (red color). The atoms with more electronegativity including O, F, and N show negative potential regions with blue color.

#### In silico* ADME* evaluation

The physiochemical and pharmacokinetic properties for all 6-nitroquinazoline conjugated with 1,3,4-thiadiazole and diaryl-urea **8a-l** were computed using Swiss *ADME* online (http://www.swissadme.ch/index.php) toolkit [69]. Through this in silico study under the Lipniski’s rule, several determined drug-likeness parameters were compared with the known drugs [70–74]. These evaluated parameters are summarized in Table 5. In terms of drug likeness properties, it can be seen that there is not any violation to Lipinski rules for these compounds. Consensus Log *P*_o/w_ is predicted in the range of 2.23–3.85. The calculations predict no Blood Brain Barrier (BBB).

**Table 5:** Drug likeness properties of ligands **8a-l**

Ligand	MW	TPSA	GI	BBB	MlogP	Lipinski	N rotatable bond	N H-bond acceptor	N H-bond donor	C Log *P*_o/w_
**8a**	425.44	192.05	Low	No	1.48	Yes	7	7	2	2.24
**8b**	439.47	192.05	Low	No	1.71	Yes	7	7	2	2.64
8c	439.47	192.05	Low	No	1.71	Yes	7	7	2	2.64
**8d**	455.47	201.28	Low	No	1.22	Yes	8	8	2	2.23
**8e**	455.47	201.28	Low	No	1.22	Yes	8	8	2	2.29
**8f**	443.43	192.05	Low	No	1.86	Yes	7	8	2	2.60
**8g**	459.89	192.05	Low	No	1.98	Yes	7	7	2	2.84
**8h**	459.89	192.05	Low	No	1.98	Yes	7	7	2	2.79
**8i**	504.34	192.05	Low	No	2.10	Yes	7	7	2	2.88
**8j**	494.33	192.05	Low	No	2.48	Yes	7	7	2	3.29
**8k**	473.92	192.05	Low	No	2.21	Yes	7	7	2	3.13
**8l**	527.89	192.05	Low	No	2.82	Yes	8	10	2	3.85

#### Molecular docking studies

**Fig. 6.**
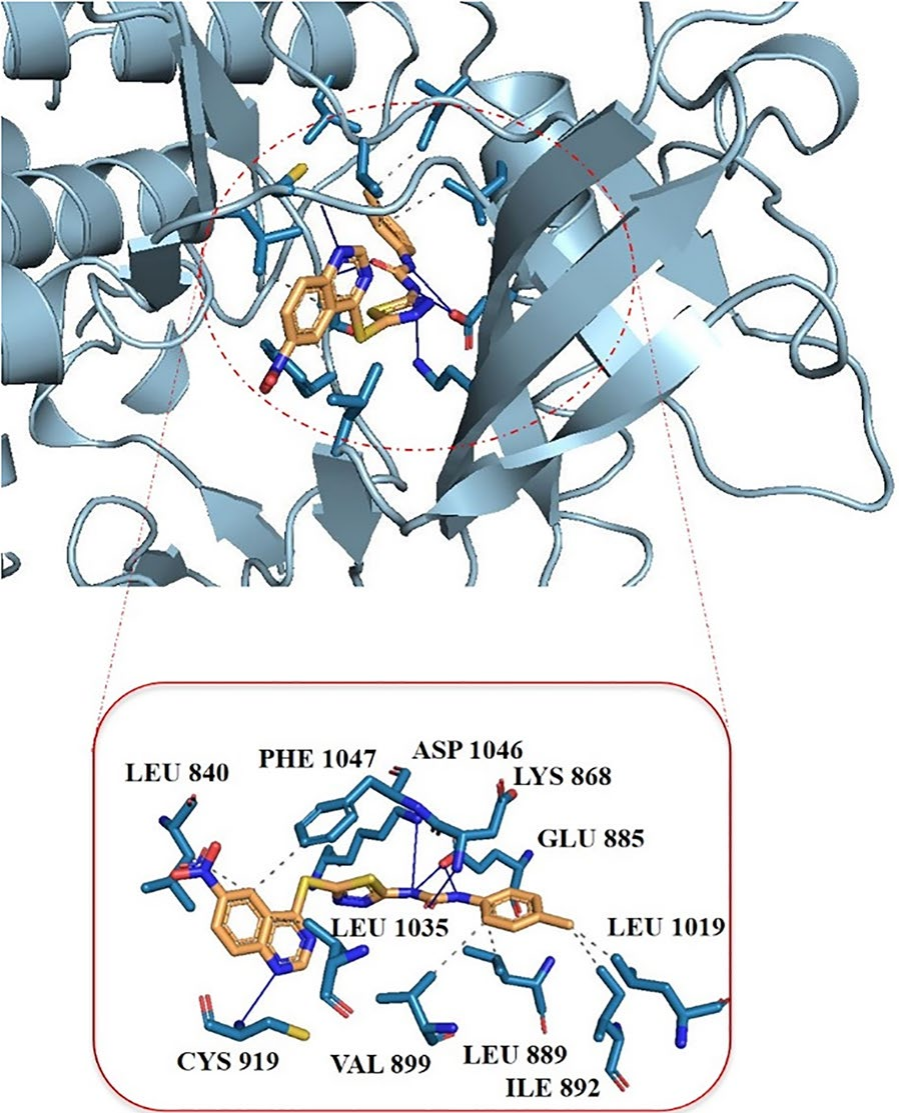
3dimensional (3D) binding interactions of compound **8c**. The hydrophobic interactions and hydrogen bonds are indicated as black dotted and blue lines, respectively

To investigate the interactions between **8c** and VEGFR2 target, the molecular docking calculation was performed. There are two types of interactions between SM1 ligand and target VEGFR2. The ligand forms hydrophobic interactions with LEU 840, LEU 889, ILE 892, VAL 899, LEU 1019, LEU 1035, PHE 1047 and hydrogen bonds with LYS 868, GLU 885, CYS 919, and ASP 1046 (Fig. 6).

**Fig. 7:**
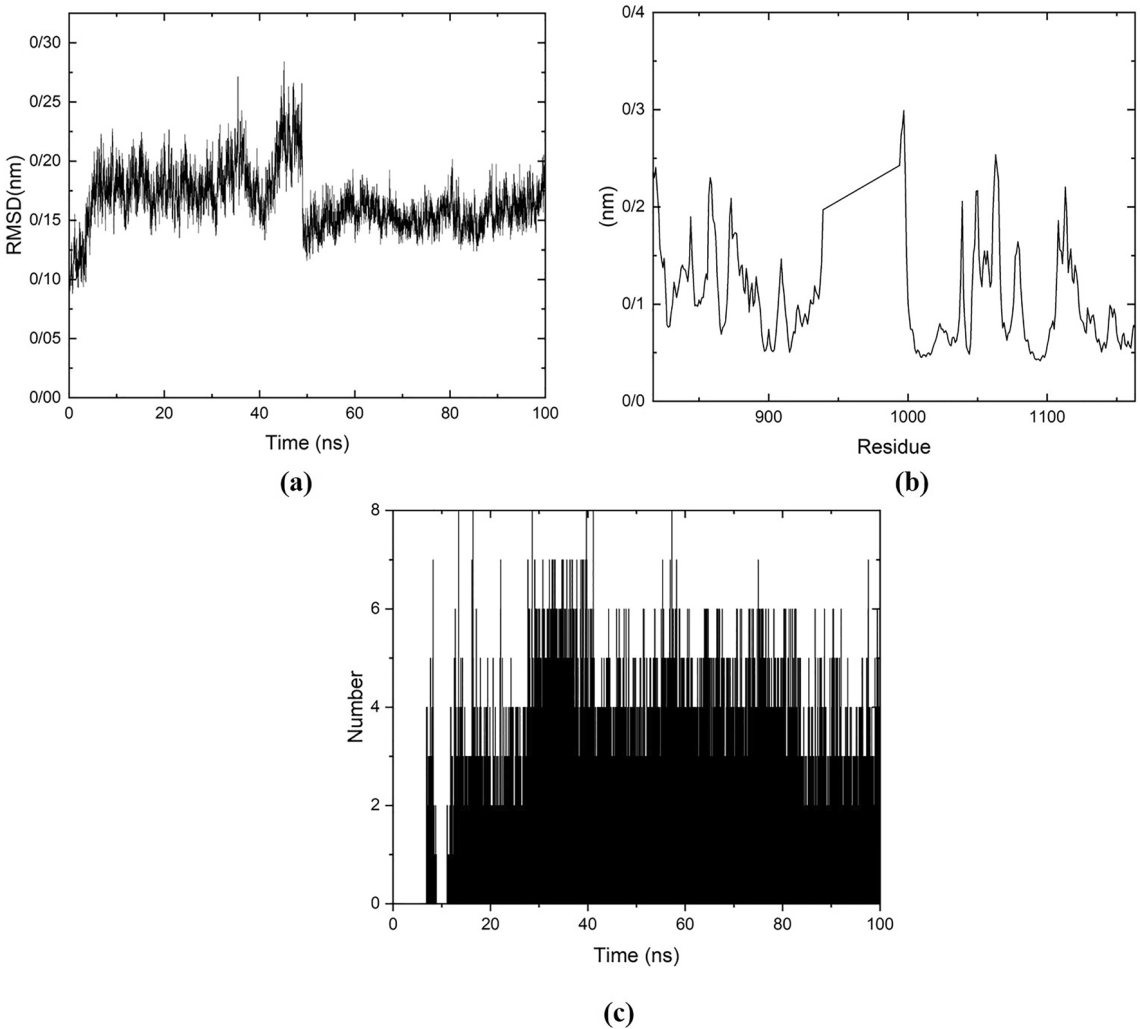
RMSD plot for the backbone atoms (**a**), RMSF plot of the complex (**b**), and the number of hydrogen bonds between the protein target VEGFR2 and compound **8c**

To assess the stability of the complex including protein and ligand, an MD simulation was performed for 100 ns. It can be seen that, in Fig. 7, the number of hydrogen bonds between protein and ligand vary in the range of 1–4 in most of the time of the simulation. The RMSD plot which is an indicator of stability of the protein, remains stable 0.15 nm after 50 ns. RMSF, which is used to evaluate the mobility of the residues, show low fluctuations and the structure remains stable during simulation time, the peaks are related to the residues out of the active site of protein. Thus, according to the results of RMSD, RMSF, and H-bond interactions, we can see that the structure remains stable during the 100 ns.

## Conclusion

A novel series of 1-(5-((6-nitroquinazoline-4-yl)thio)-1,3,4-thiadiazol-2-yl)-3-phenylurea derivatives **8a–l** were designed, synthesized, investigated for their potential as an anticancer agent against A549, MDA-MB231, and MCF7. Among them, compound **8b** and **8c** bearing methyl group at C-3 and C-4 positions of phenyl ring showed good in vitro anti-proliferative potency with IC_50_ value of 29.0 ± 0.005 µM and 25.0 ± 0.001 µM against MCF7, respectively, which was comparable with Etoposide (IC_50_ = 29.8 ± 0.0007 µM). The *ADME* properties were calculated to assess the drug-like features. The quantitative electrostatic potential analysis was done to have a better understanding of the relationship between the electronical structure and activity. The stability of the protein–ligand complex was verified through 100 ns MD simulation. Finally, considering better cytotoxicity of compound **8c**, the molecular docking study for this ligand was performed, showing several hydrogen bonds, hydrophobic interactions, and van der Waals forces between it and residues in the active sites of VEGFR-2. Although it has been anticipated that these compounds possess great anti-proliferative potencies, this goal was not achieved which might be related to the adverse role of the nitro group. Therefore, changing this functionality in an attempt to find further potent anticancer agents could be an interesting goal for future studies.

### Supplementary Information

**Additional file 1.** Additional file 1 of Novel quinazolines bearing 1,3,4-thiadiazole-aryl urea derivative as anticancer agents: Design, Synthesis, Molecular docking, DFT and Bioactivity evaluations

## Data Availability

The authors confirm that the data supporting the finding of this study are available within the manuscript.
