# Supplementary material for: Novel quinazolines bearing 1,3,4-thiadiazole-aryl urea derivative as anticancer agents: design, synthesis, molecular docking, DFT and bioactivity evaluations
Source: BMC Chem. 2024 Feb 12;18(1):30. doi: 10.1186/s13065-024-01119-0 (PMC10863284; doi:10.1186/s13065-024-01119-0)

Supporting Information

Novel quinazolines bearing 1,3,4-thiadiazole-aryl urea derivative as anticancer agents: Design, Synthesis, Molecular docking, DFT and Bioactivity evaluations

Sara Masoudinia [a], Marjaneh Samadi Zadeh [a], Maliheh Safavi [b], Hamid Reza Bijanzadeh [c], Alireza Foroumadi [d,f,*]

[a] Department of Chemistry, Islamic Azad University, Central Tehran Branch, Tehran, Iran

[b] Department of Biotechnology, Iranian Research Organization for Science and Technology (IROST), Tehran, Iran

[c] Department of Chemistry, Tarbiat Modares University, Tehran, Iran

[d] Department of Medicinal Chemistry, Faculty of Pharmacy, Tehran University of Medical Sciences, Tehran, Iran

[f] Drug Design and Development Research Center, The Institute of Pharmaceutical Sciences (TIPS), Tehran University of Medical Sciences, Tehran, Iran

**List of contents**	Page
Title, author's name address and Table	1,2
^{1}H NMR spectrum of **8a**	3
^{13}C NMR spectrum of **8a**	4
^{1}H NMR spectrum of **8b**	5
^{13}C NMR spectrum of **8b**	6
^{1}H NMR spectrum of **8c**	7
^{13}C NMR spectrum of **8c**	8
^{1}H NMR spectrum of **8d**	9
^{13}C NMR spectrum of **8d**	10
^{1}H NMR spectrum of **8e**	11
^{13}C NMR spectrum of **8e**	12
^{1}H NMR spectrum of **8f**	13
^{13}C NMR spectrum of **8f**	14
^{1}H NMR spectrum of **8g**	15
^{13}C NMR spectrum of **8g**	16
^{1}H NMR spectrum of **8h**	17
^{13}C NMR spectrum of **8h**	18

^{1}H NMR spectrum of **8i**	19
^{13}C NMR spectrum of **8i**	20
^{1}H NMR spectrum of **8j**	21
^{13}C NMR spectrum of **8j**	22
^{1}H NMR spectrum of **8k**	23
^{13}C NMR spectrum of **8k**	24
^{1}H NMR spectrum of **8l**	25
^{13}C NMR spectrum of **8l**	26

^{1}H NMR spectrum of 1-(5-((6-Nitroquinazolin-4-yl)thio)-1,3,4-thiadiazol-2-yl)-3-phenylurea (8a)

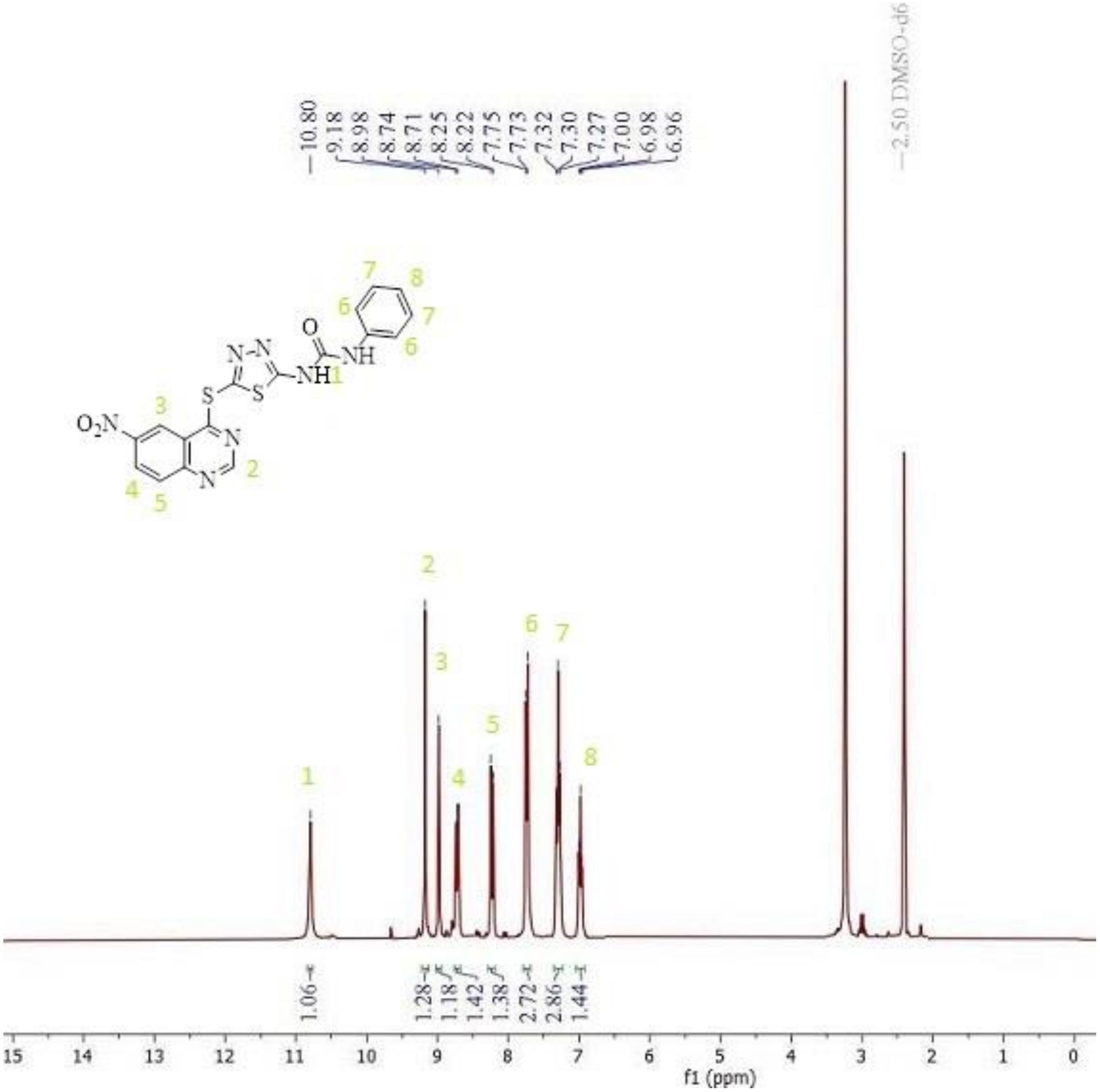

^{13}C NMR spectrum of 1-(5-((6-Nitroquinazolin-4-yl)thio)-1,3,4-thiadiazol-2-yl)-3-phenylurea (8a)

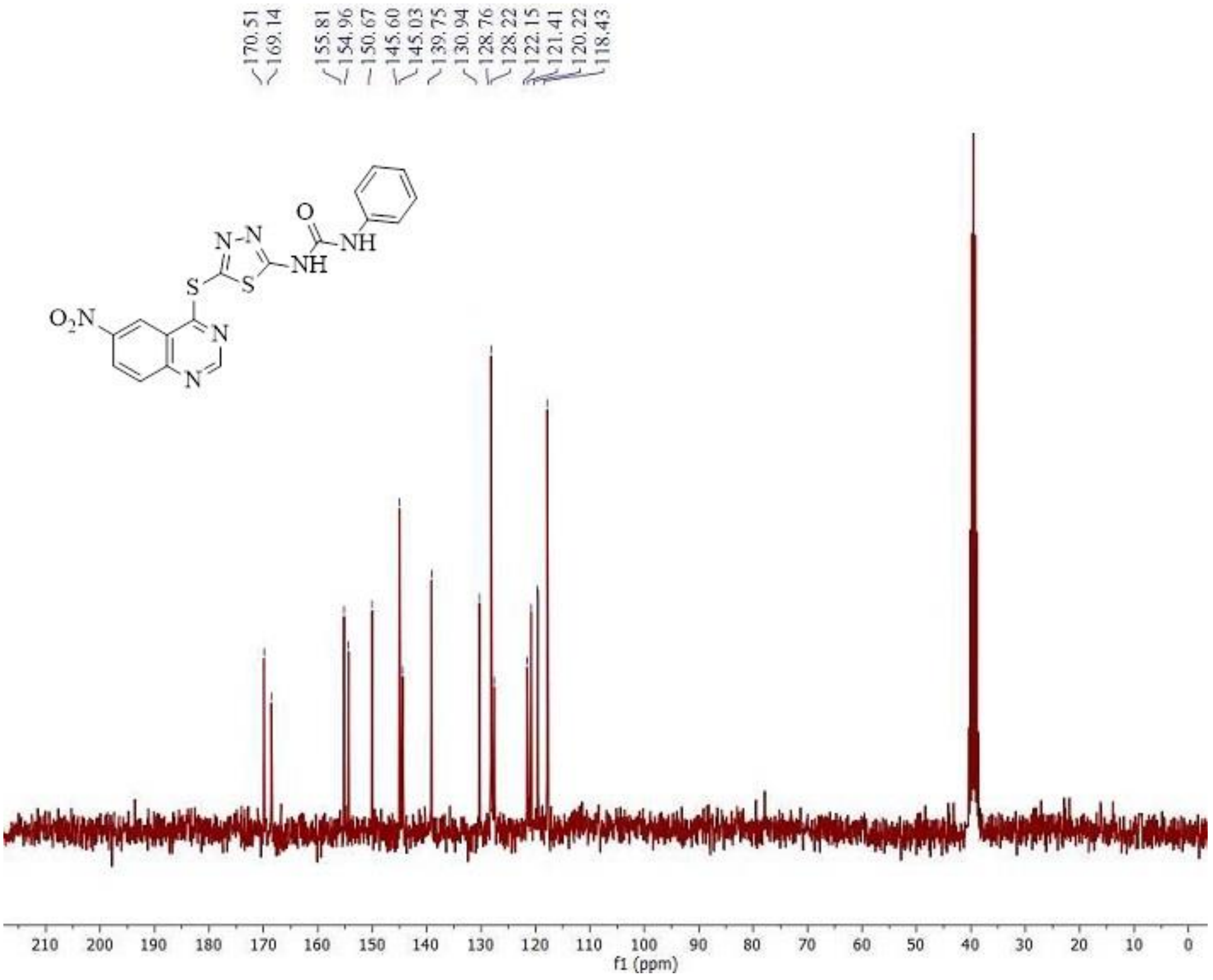

^{1}H NMR spectrum of 1-(5-((6-Nitroquinazolin-4-yl)thio)-1,3,4-thiadiazol-2-yl)-3-(m-tolyl)urea (8b)

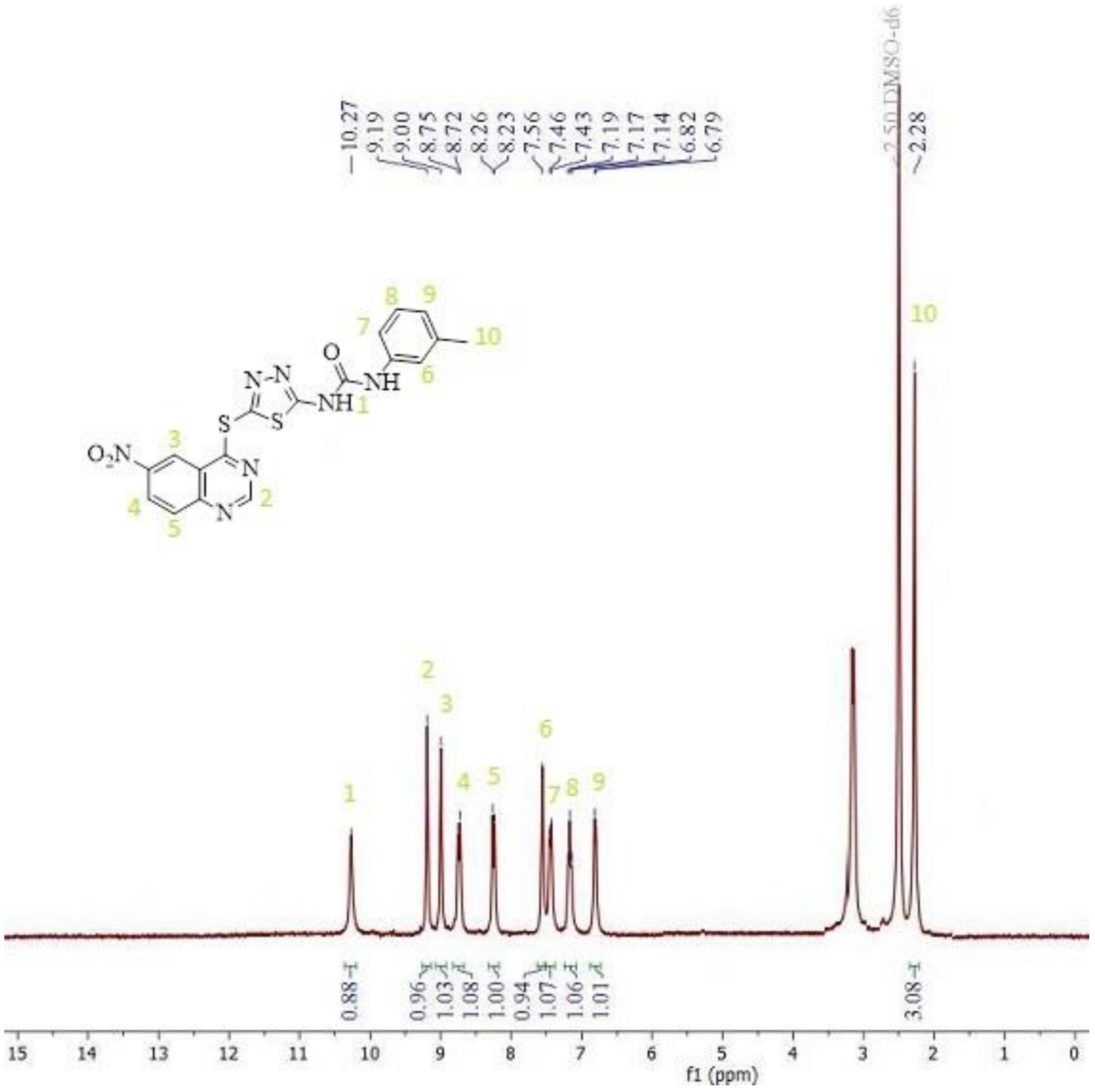

^{13}C NMR spectrum of 1-(5-((6-Nitroquinazolin-4-yl)thio)-1,3,4-thiadiazol-2-yl)-3-(m-tolyl)urea (8b)

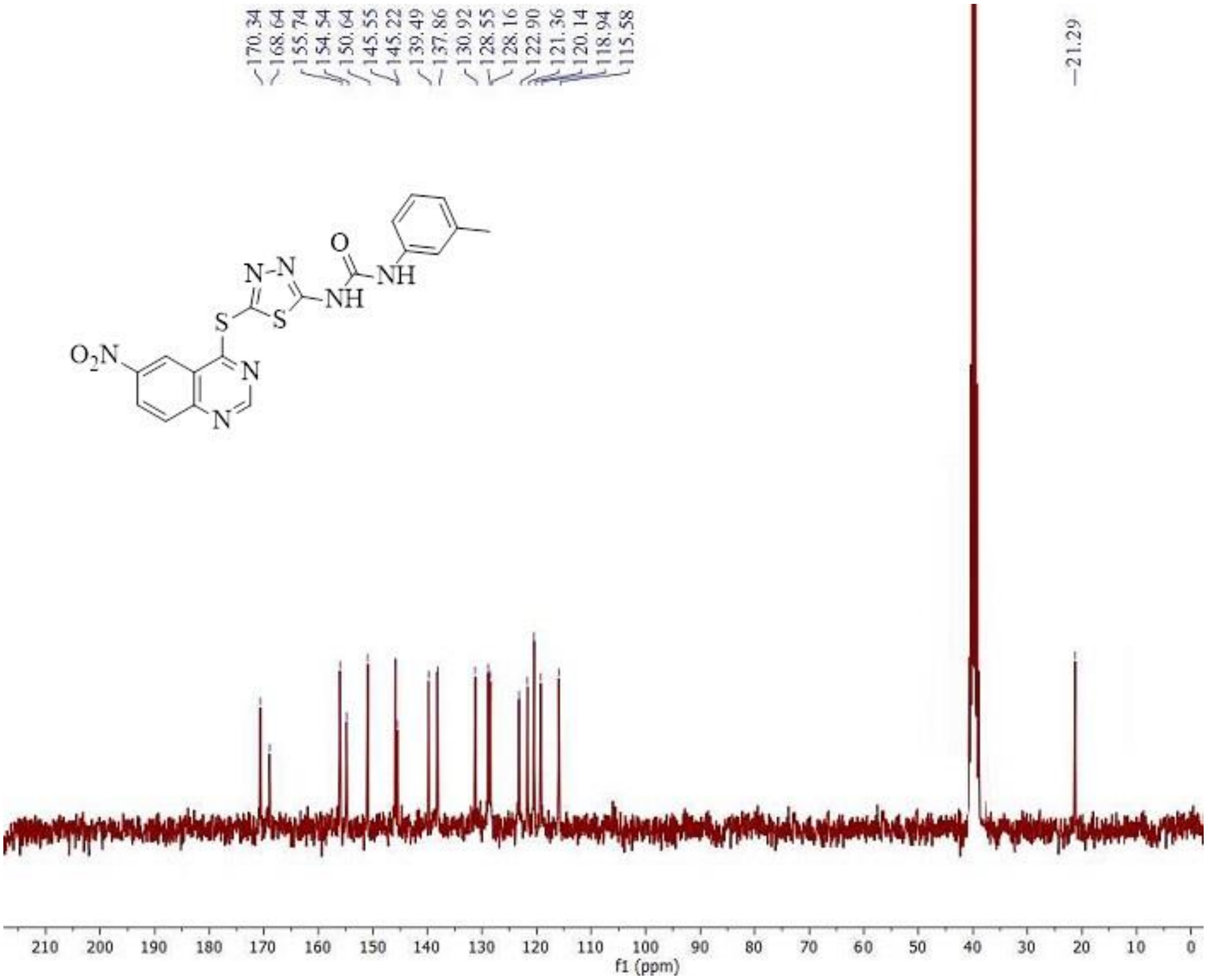

^{1}H NMR spectrum of 1-(5-((6-Nitroquinazolin-4-yl)thio)-1,3,4-thiadiazol-2-yl)-3-(p-tolyl)urea (8c)

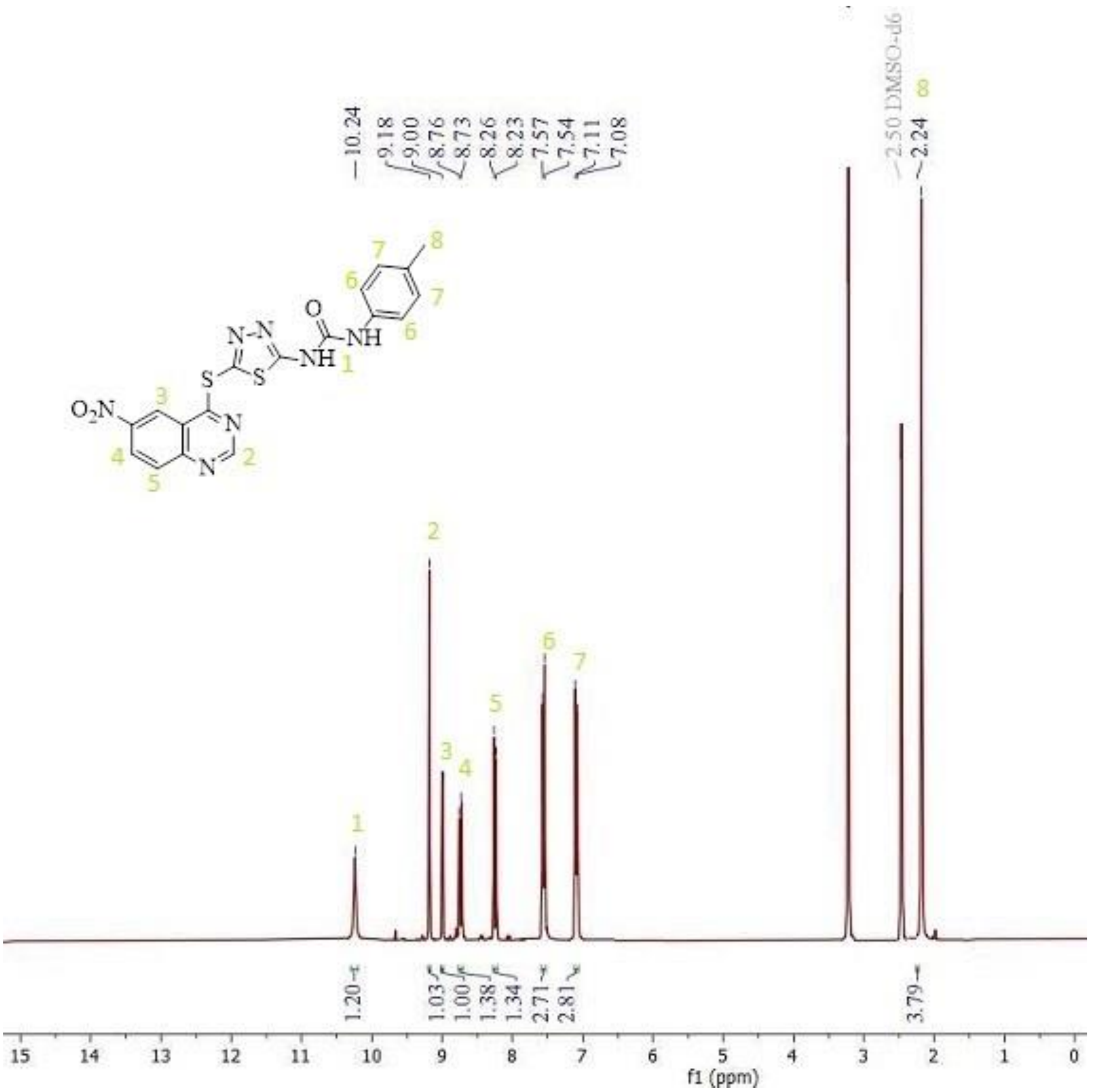

^{13}C NMR spectrum of 1-(5-((6-Nitroquinazolin-4-yl)thio)-1,3,4-thiadiazol-2-yl)-3-(p-tolyl)urea (8c)

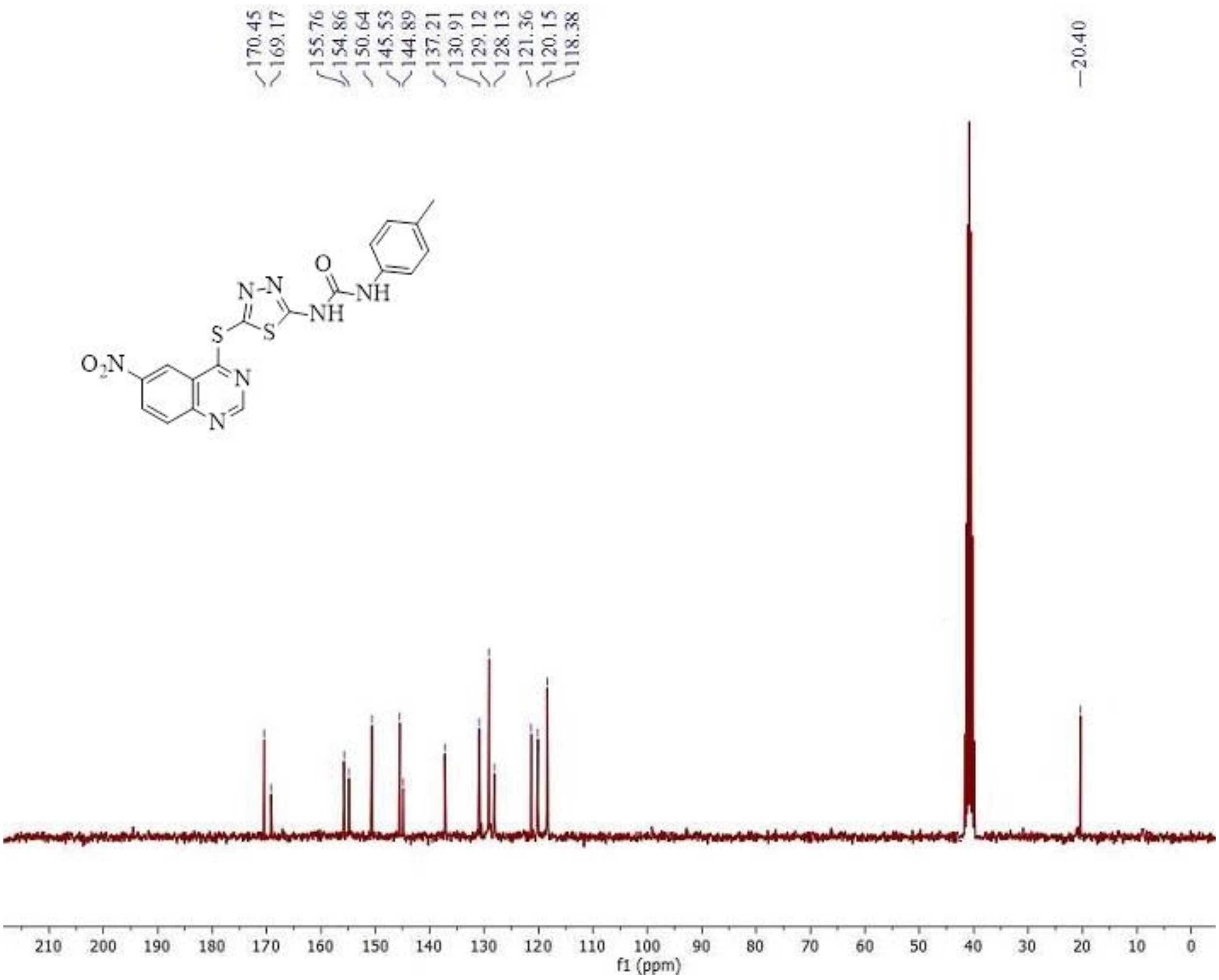

^{1}H NMR spectrum of 1-(3-Methoxyphenyl)-3-(5-((6-nitroquinazolin-4-yl)thio)-1,3,4-thiadiazol-2-yl)urea (8d)

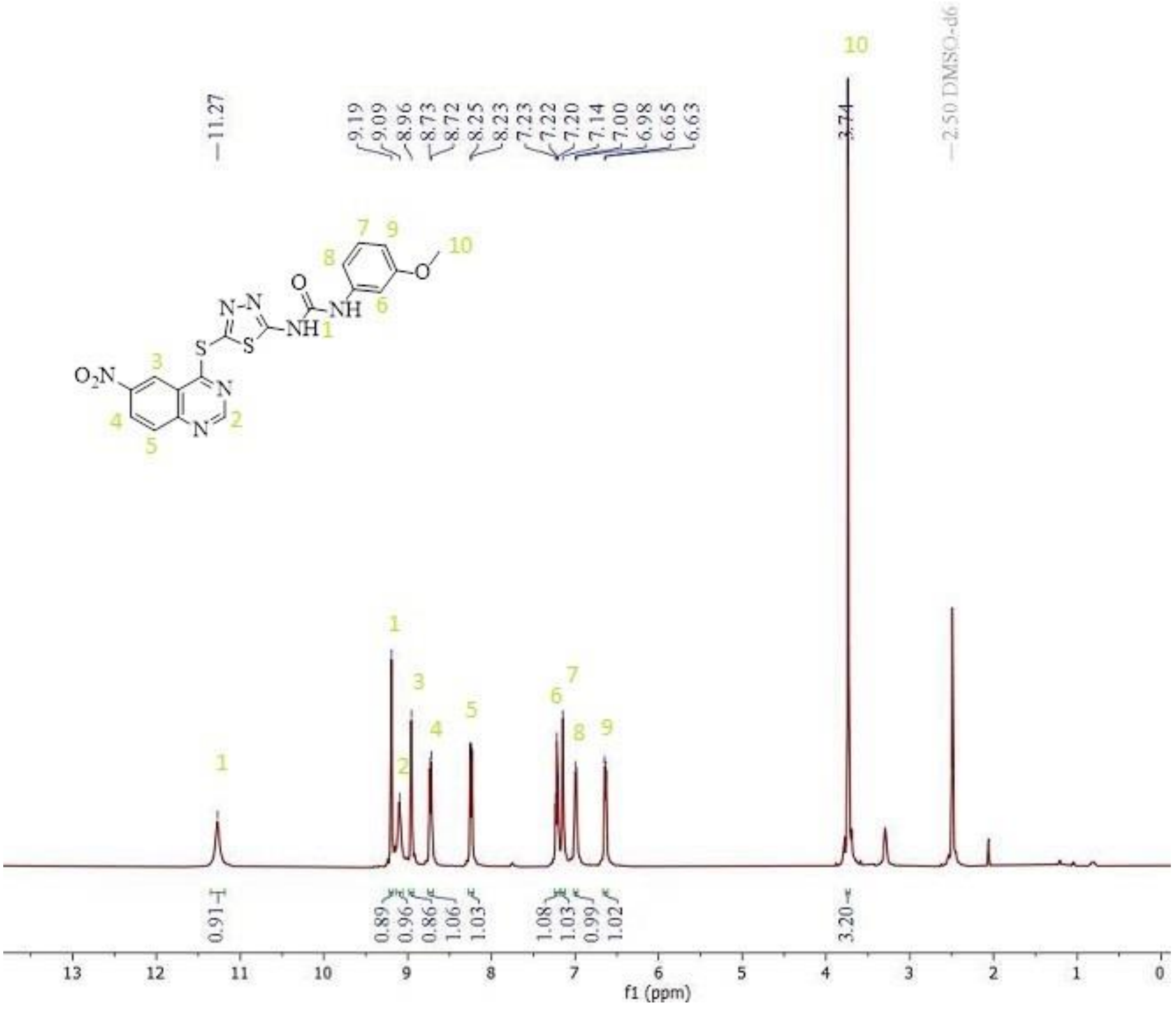

^{13}C NMR spectrum of 1-(3-Methoxyphenyl)-3-(5-((6-nitroquinazolin-4-yl)thio)-1,3,4-thiadiazol-2-yl)urea (8d)

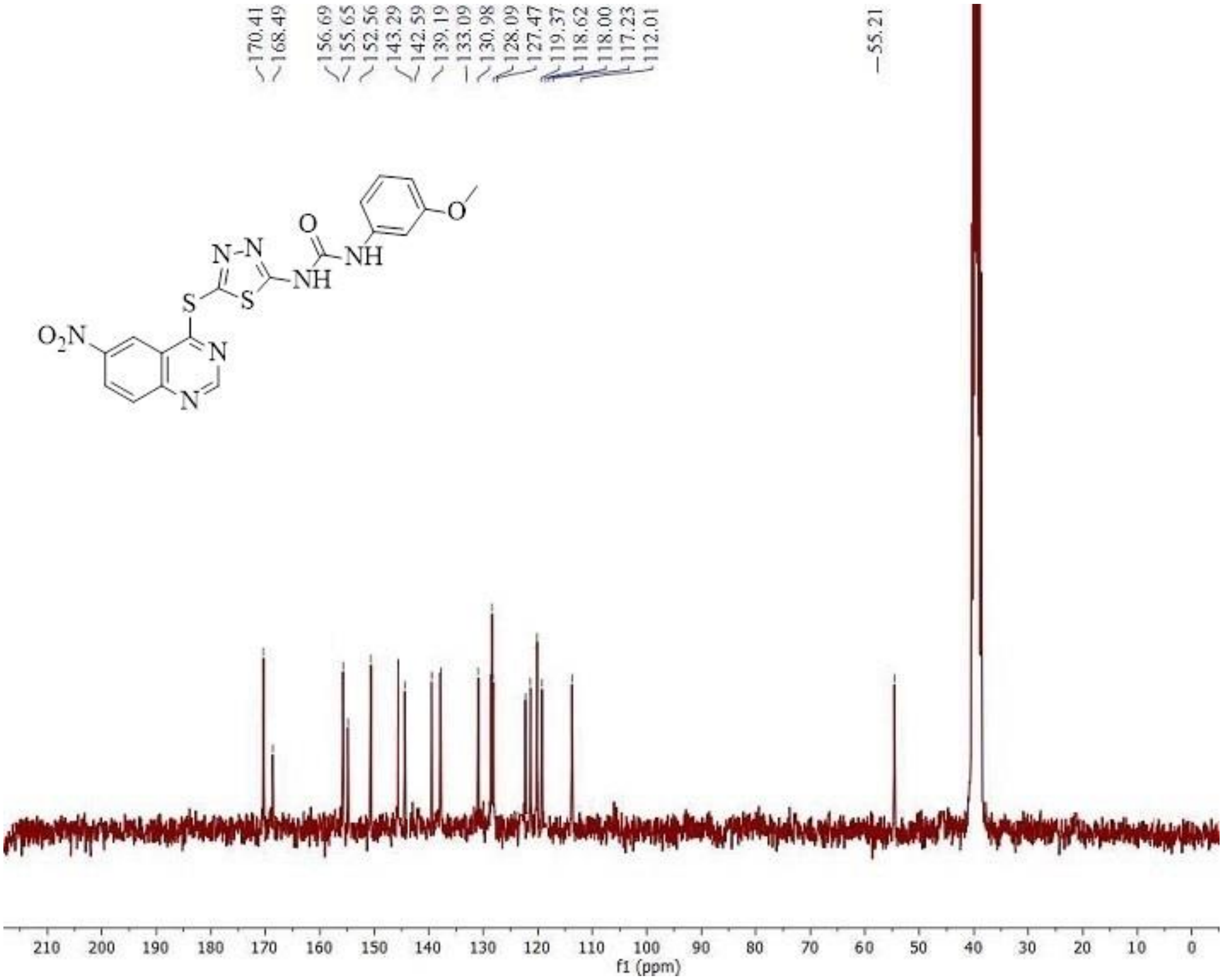

^{1}H NMR spectrum of 1-(4-Methoxyphenyl)-3-(5-((6-nitroquinazolin-4-yl)thio)-1,3,4-thiadiazol-2-yl)urea (8e)

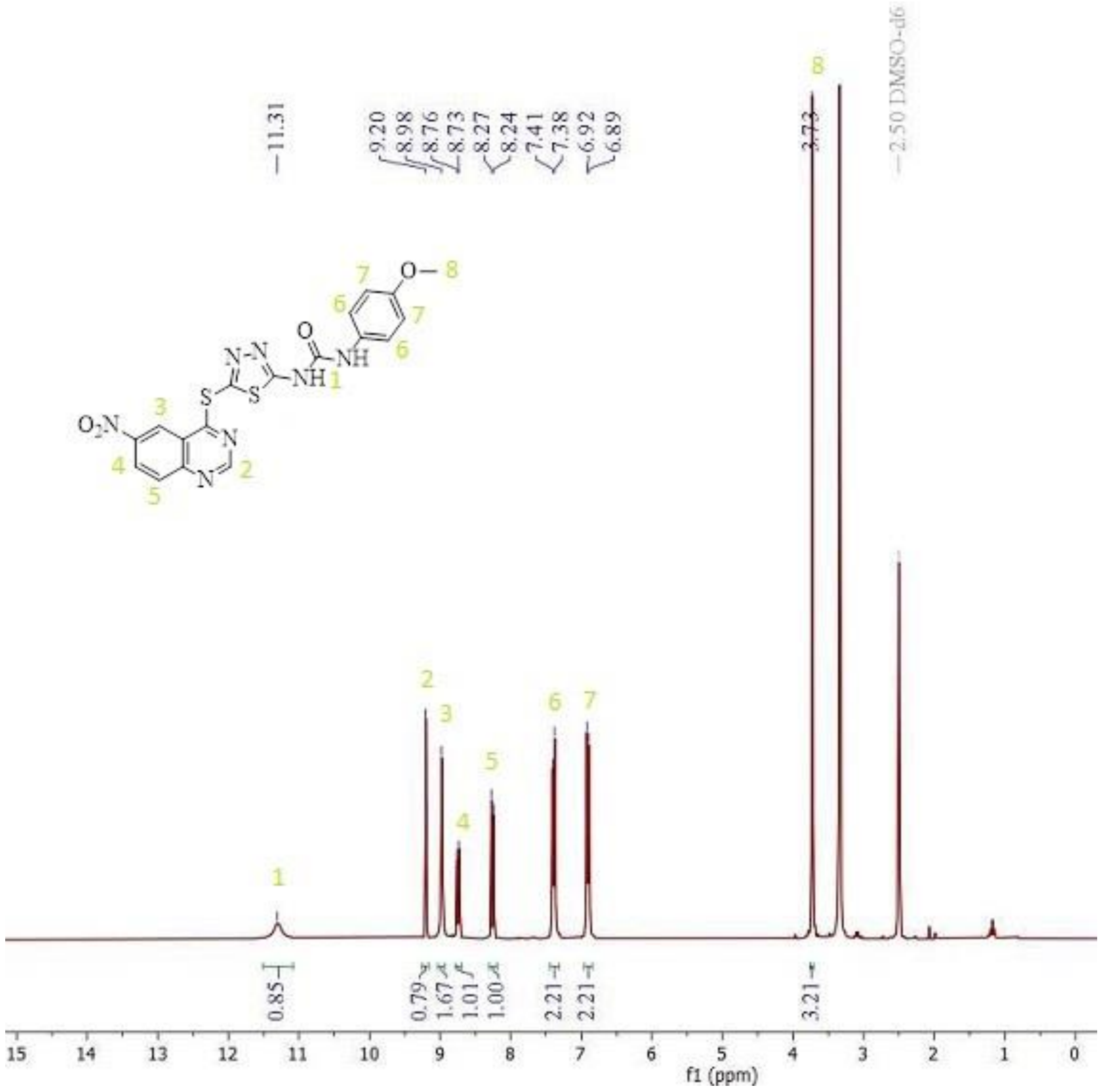

^{13}C NMR spectrum of 1-(4-Methoxyphenyl)-3-(5-((6-nitroquinazolin-4-yl)thio)-1,3,4-thiadiazol-2-yl)urea (8e)

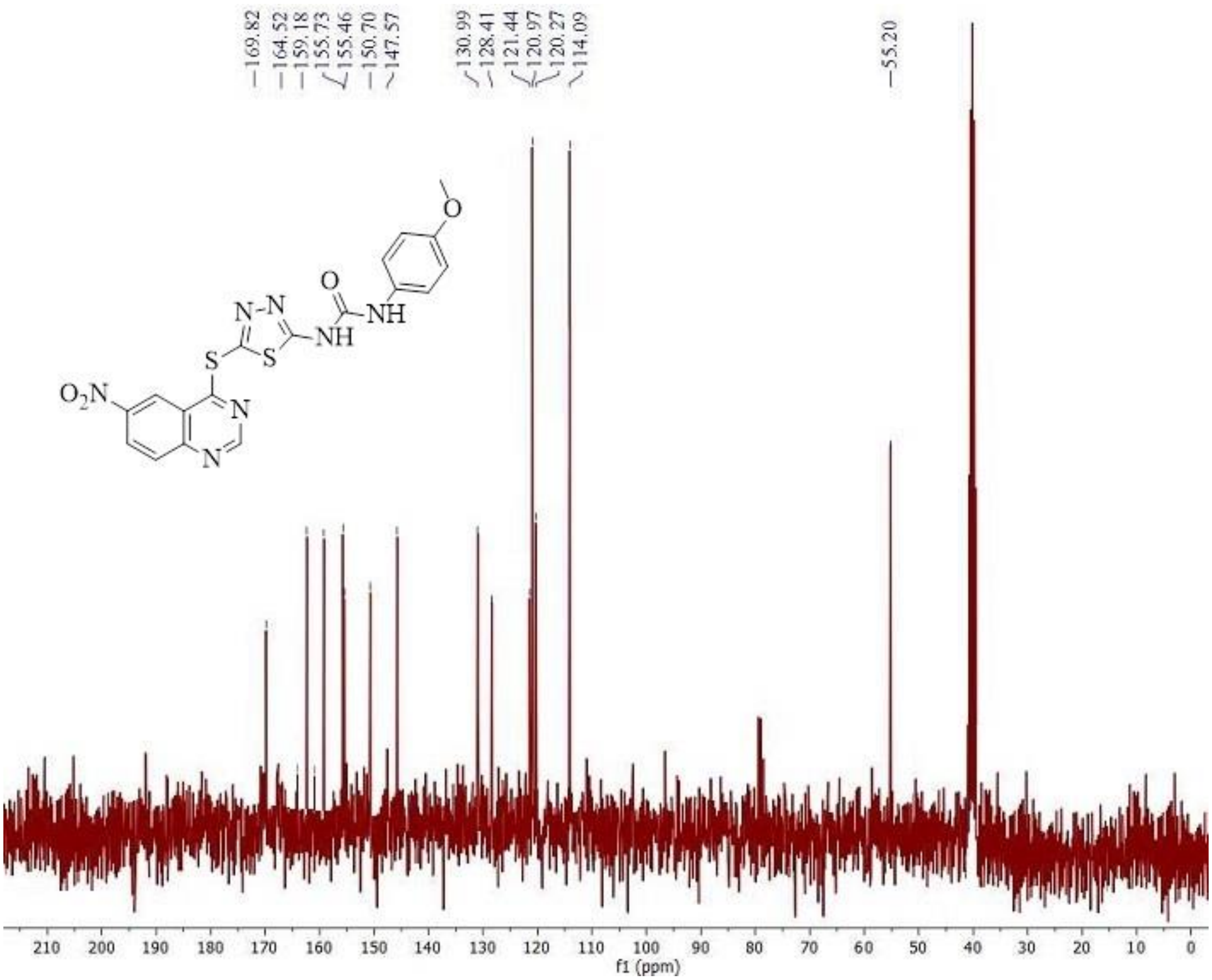

^{1}H NMR spectrum of 1-(3-Fluorophenyl)-3-(5-((6-nitroquinazolin-4-yl)thio)-1,3,4-thiadiazol-2-yl)urea (8f)

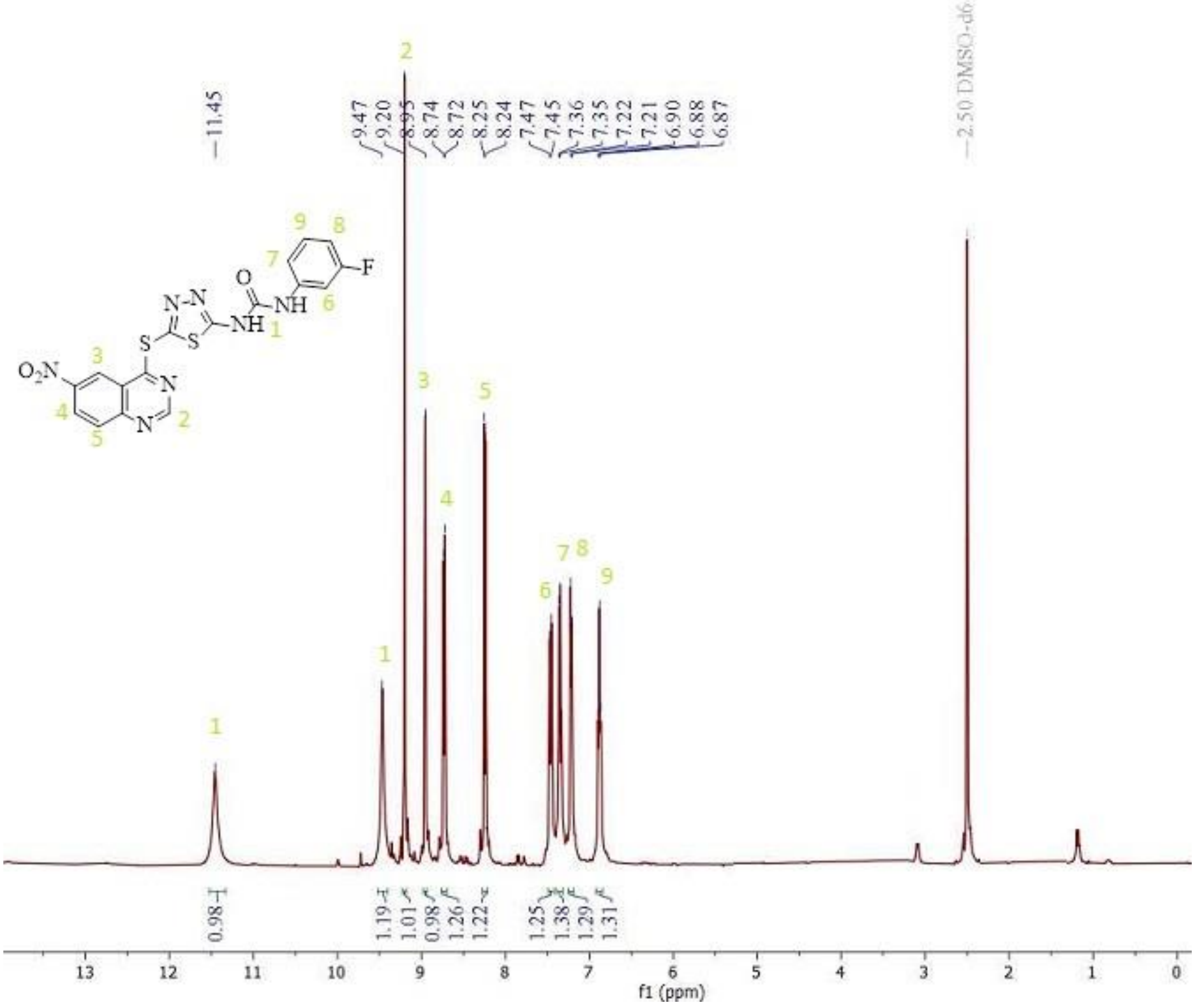

^{13}C NMR spectrum of 1-(3-Fluorophenyl)-3-(5-((6-nitroquinazolin-4-yl)thio)-1,3,4-thiadiazol-2-yl)urea (8f)

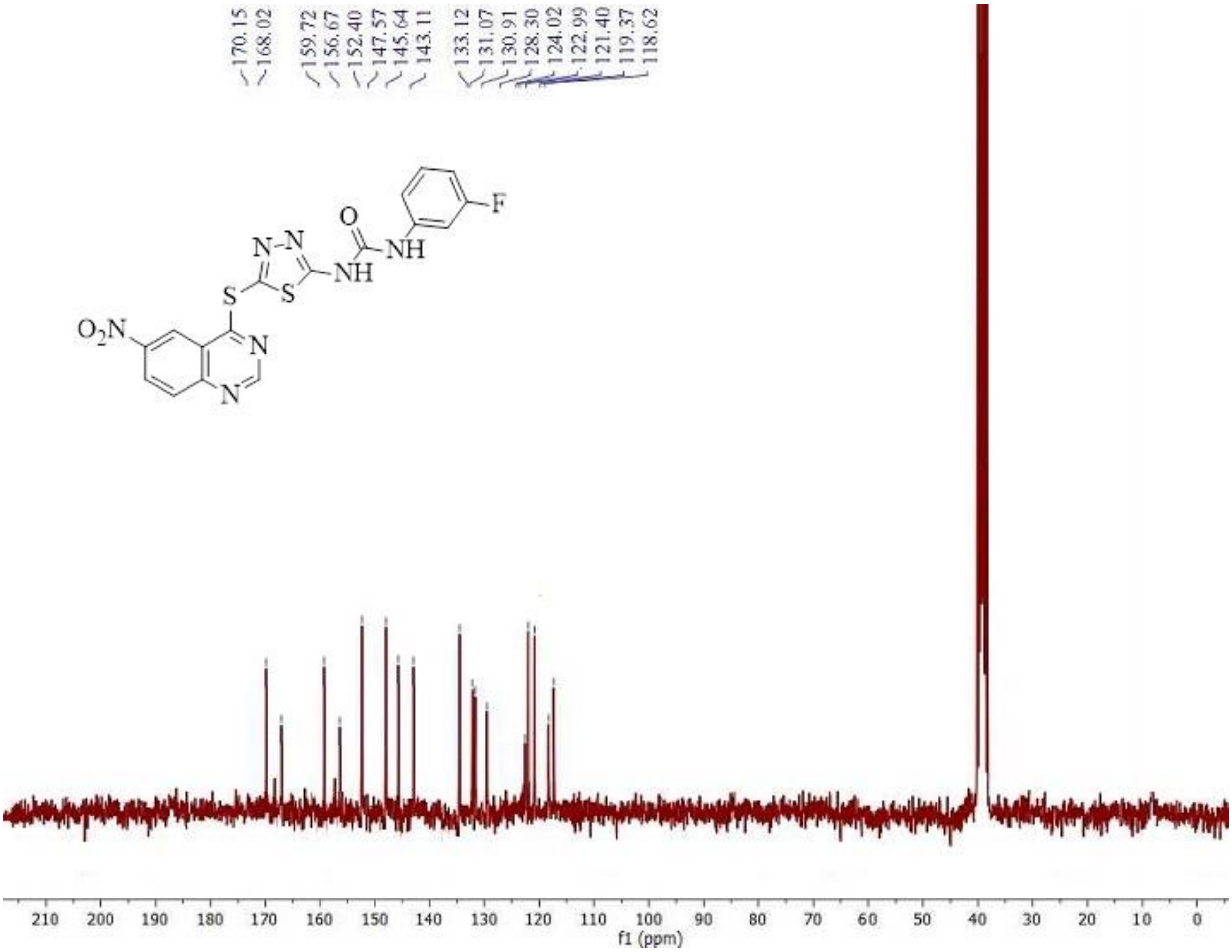

^{1}H NMR spectrum of 1-(3-Chlorophenyl)-3-(5-((6-nitroquinazolin-4-yl)thio)-1,3,4-thiadiazol-2-yl)urea (8g)

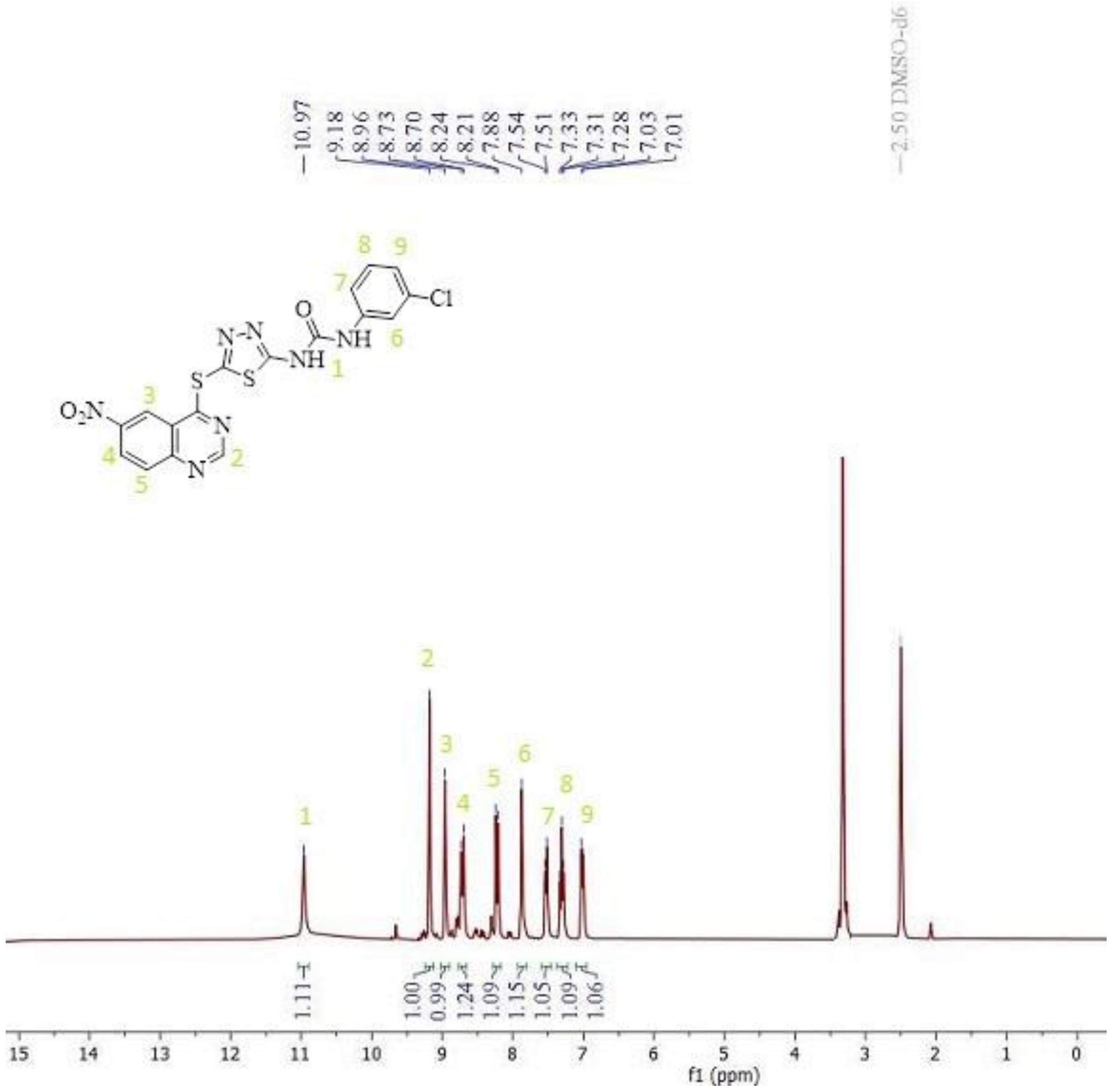

^{13}C NMR spectrum of 1-(3-Chlorophenyl)-3-(5-((6-nitroquinazolin-4-yl)thio)-1,3,4-thiadiazol-2-yl)urea (8g)

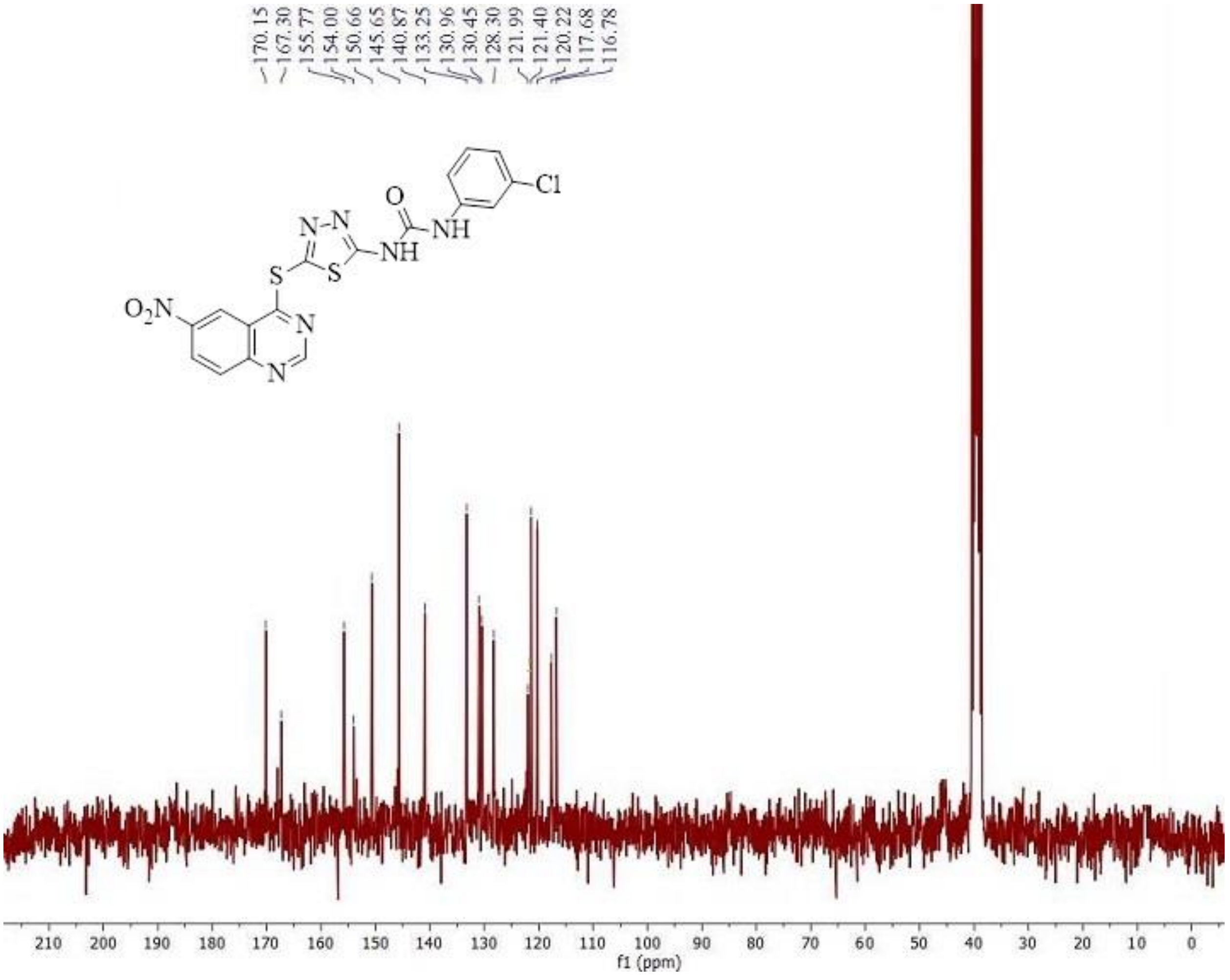

^{1}H NMR spectrum of 1-(4-Chlorophenyl)-3-(5-((6-nitroquinazolin-4-yl)thio)-1,3,4-thiadiazol-2-yl)urea (8h)

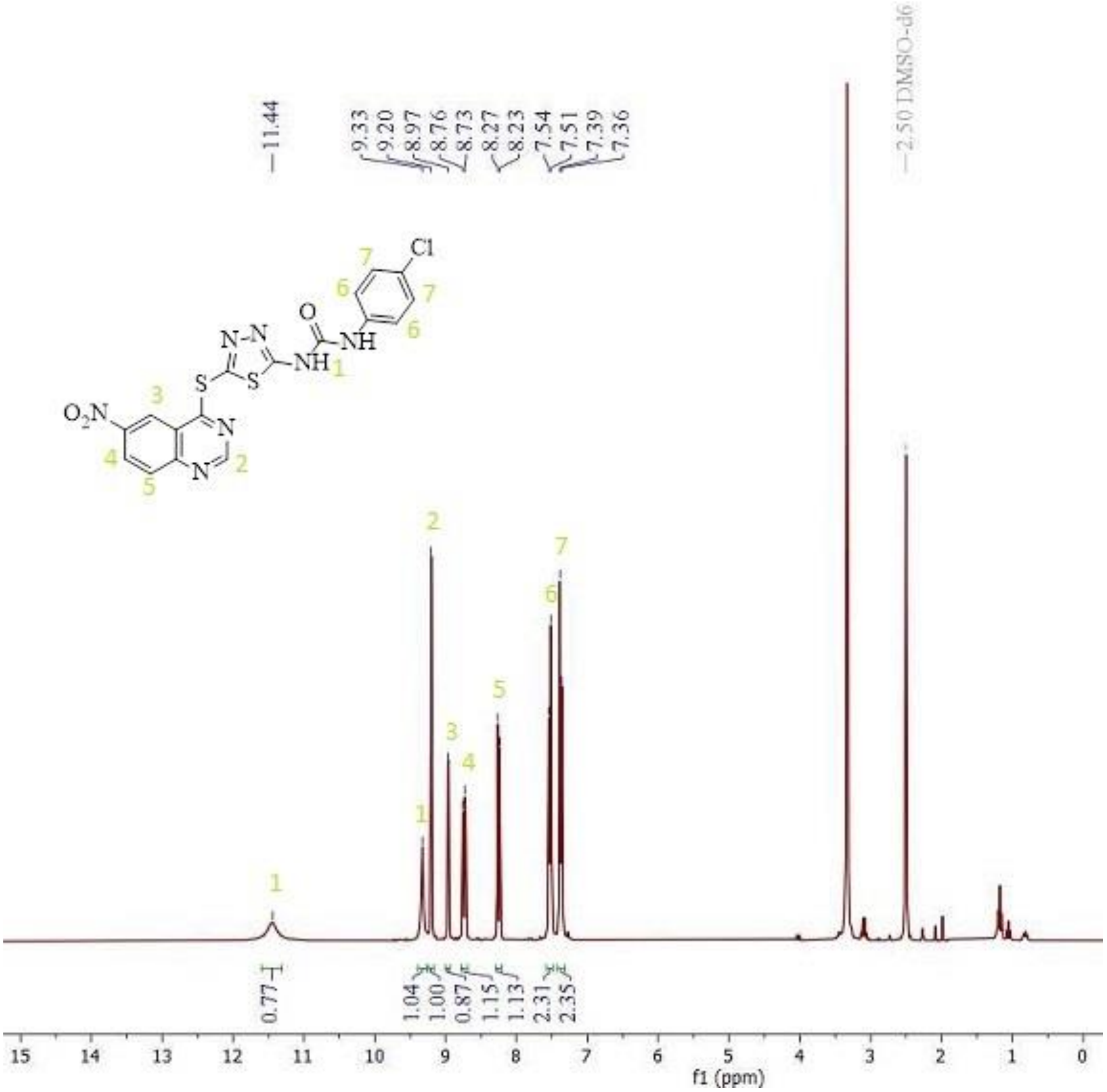

^{13}C NMR spectrum of 1-(4-Chlorophenyl)-3-(5-((6-nitroquinazolin-4-yl)thio)-1,3,4-thiadiazol-2-yl)urea (8h)

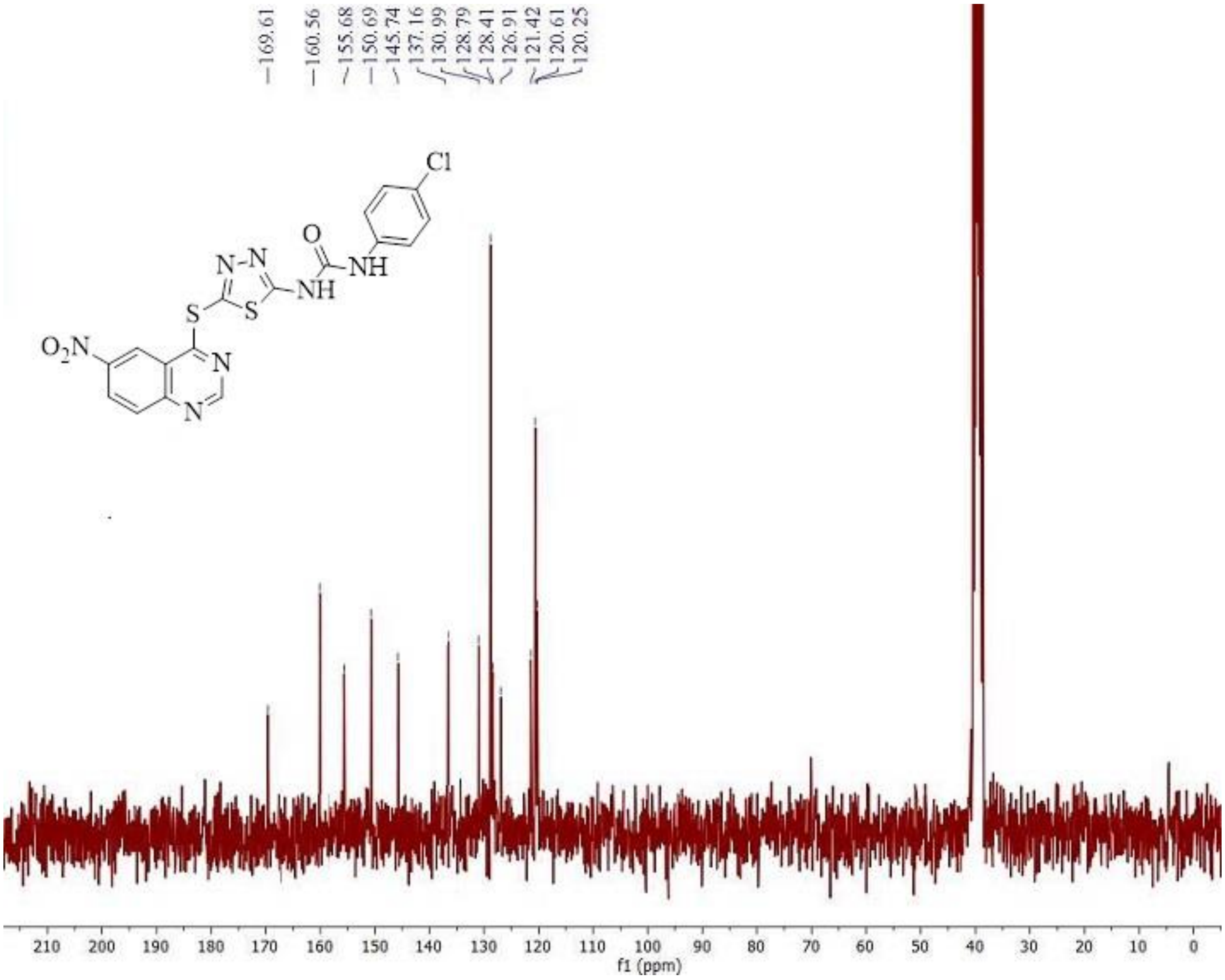

^{1}H NMR spectrum of 1-(4-Bromophenyl)-3-(5-((6-nitroquinazolin-4-yl)thio)-1,3,4-thiadiazol-2-yl)urea(8i)

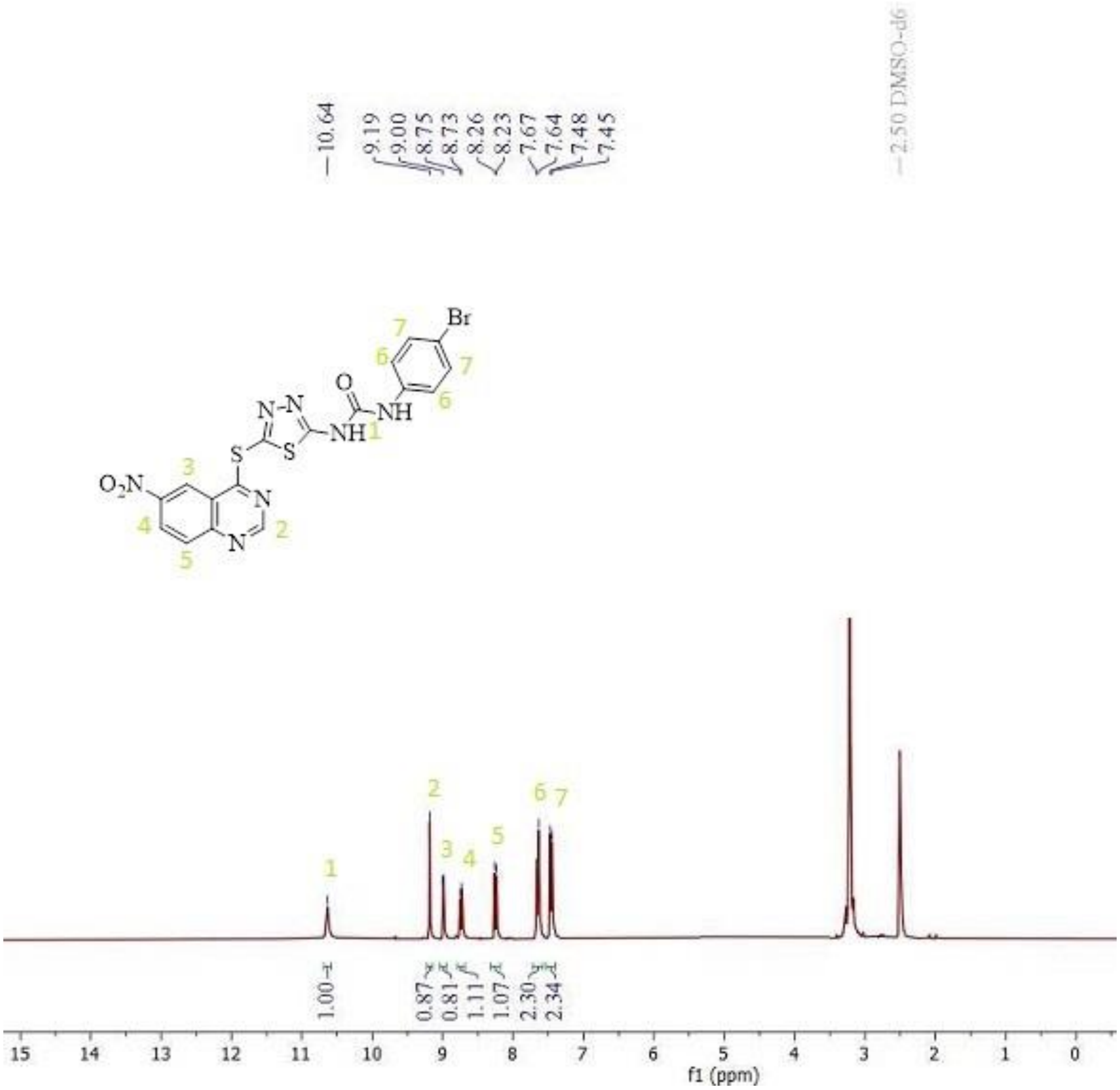

^{13}C NMR spectrum of 1-(4-Bromophenyl)-3-(5-((6-nitroquinazolin-4-yl)thio)-1,3,4-thiadiazol-2-yl)urea(8i)

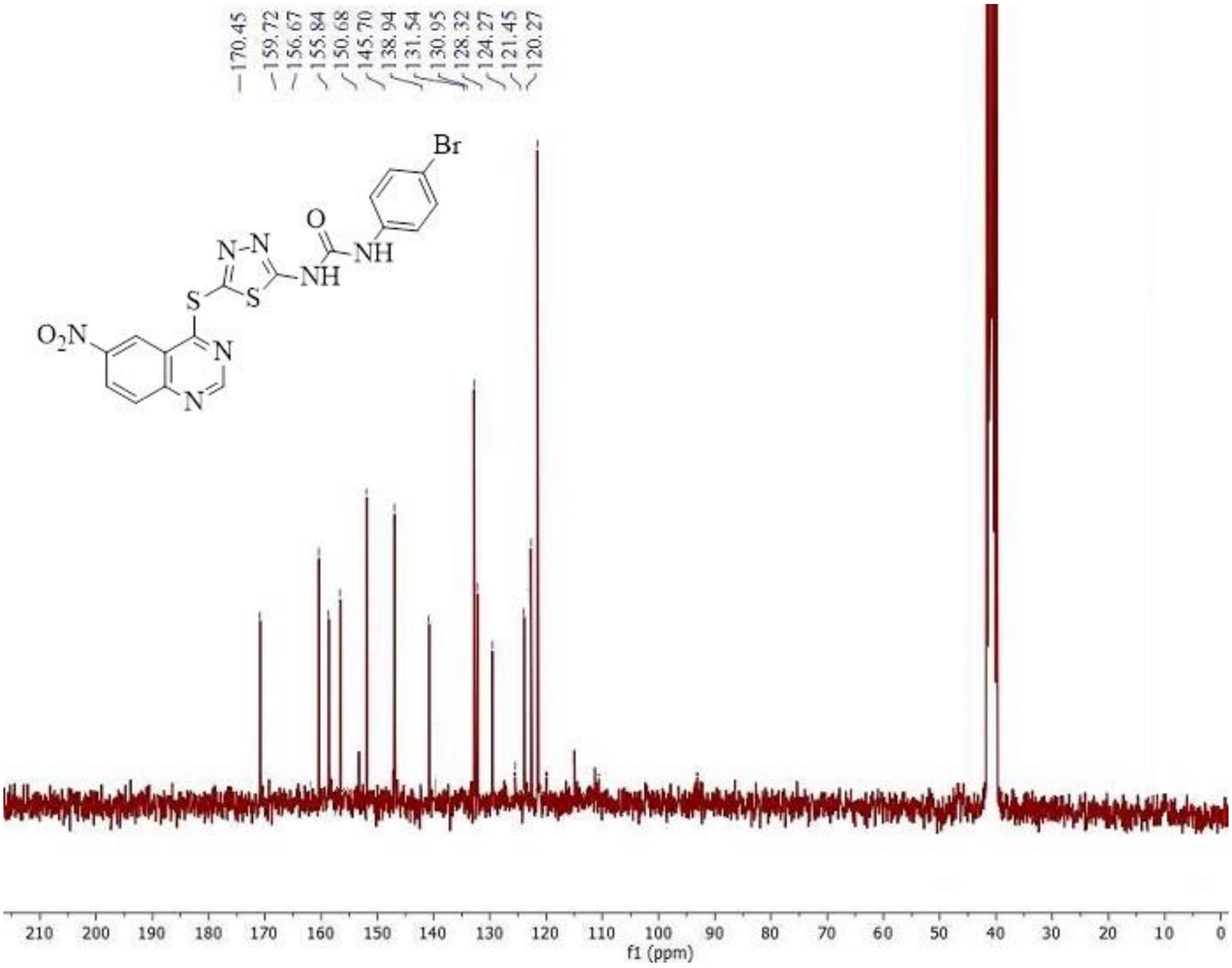

^{1}H NMR spectrum of 1-(3, 4-Dichlorophenyl)-3-(5-((6-nitroquinazolin-4-yl) thio)-1, 3, 4-thiadiazol-2-yl)urea (8j)

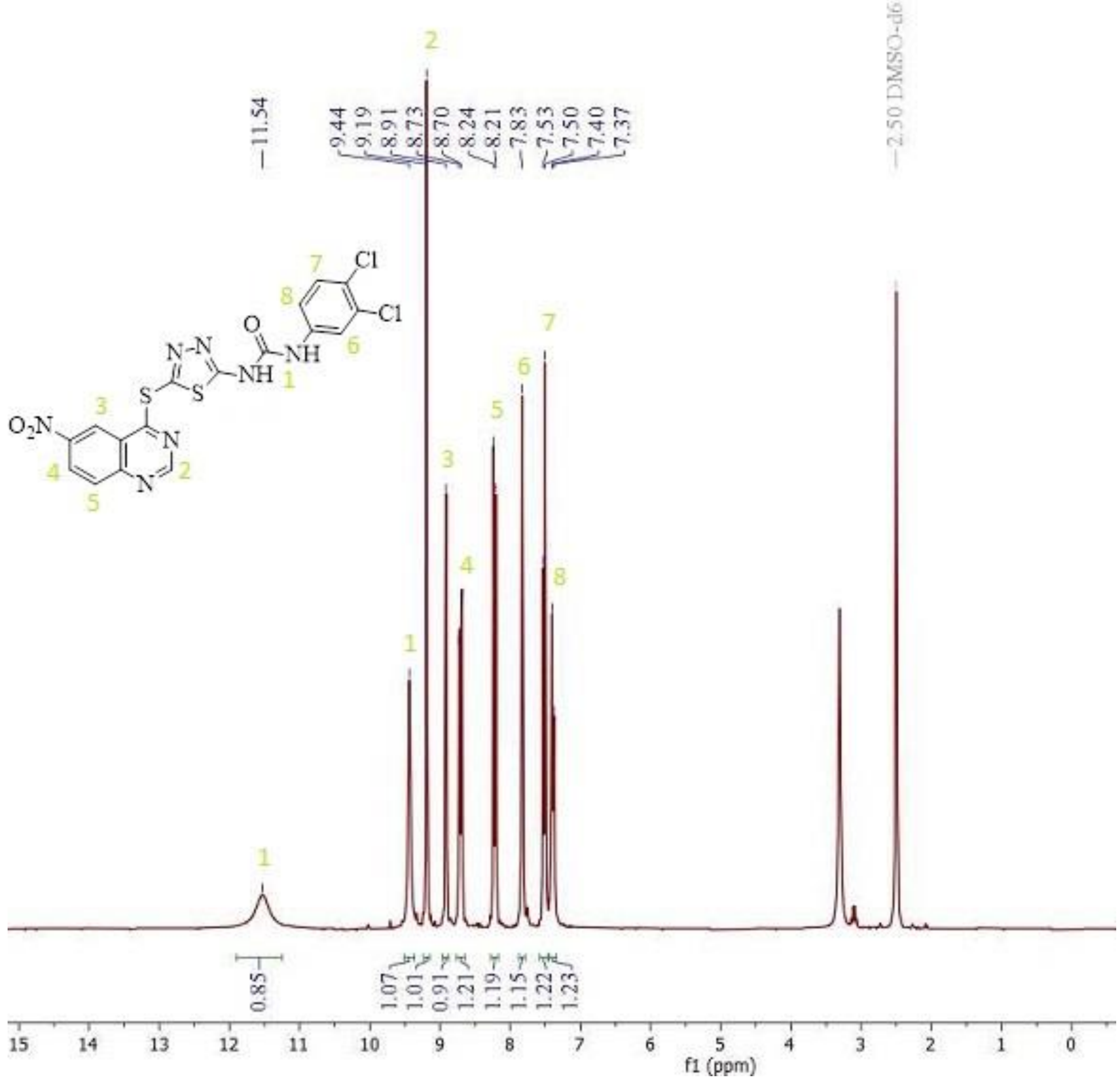

^{13}C NMR spectrum of 1-(3,4-Dichlorophenyl)-3-(5-((6-nitroquinazolin-4-yl)thio)-1,3,4-thiadiazol-2-yl)urea (8j)

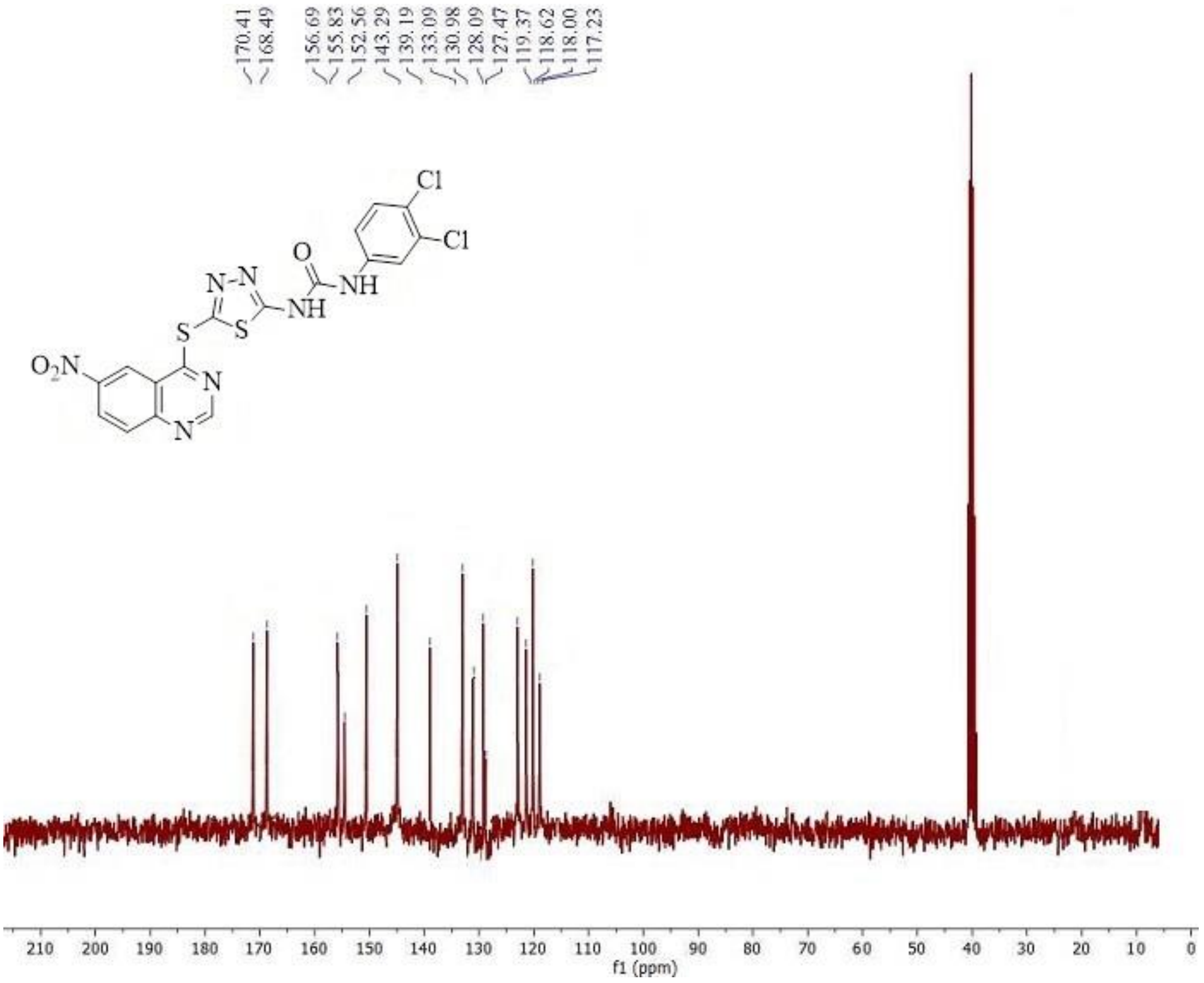

^{1}H NMR spectrum of 1-(3-Chloro-4-methylphenyl)-3-(5-((6-nitroquinazolin-4-yl)thio)-1,3,4-thiadiazol-2-yl)urea (8k)

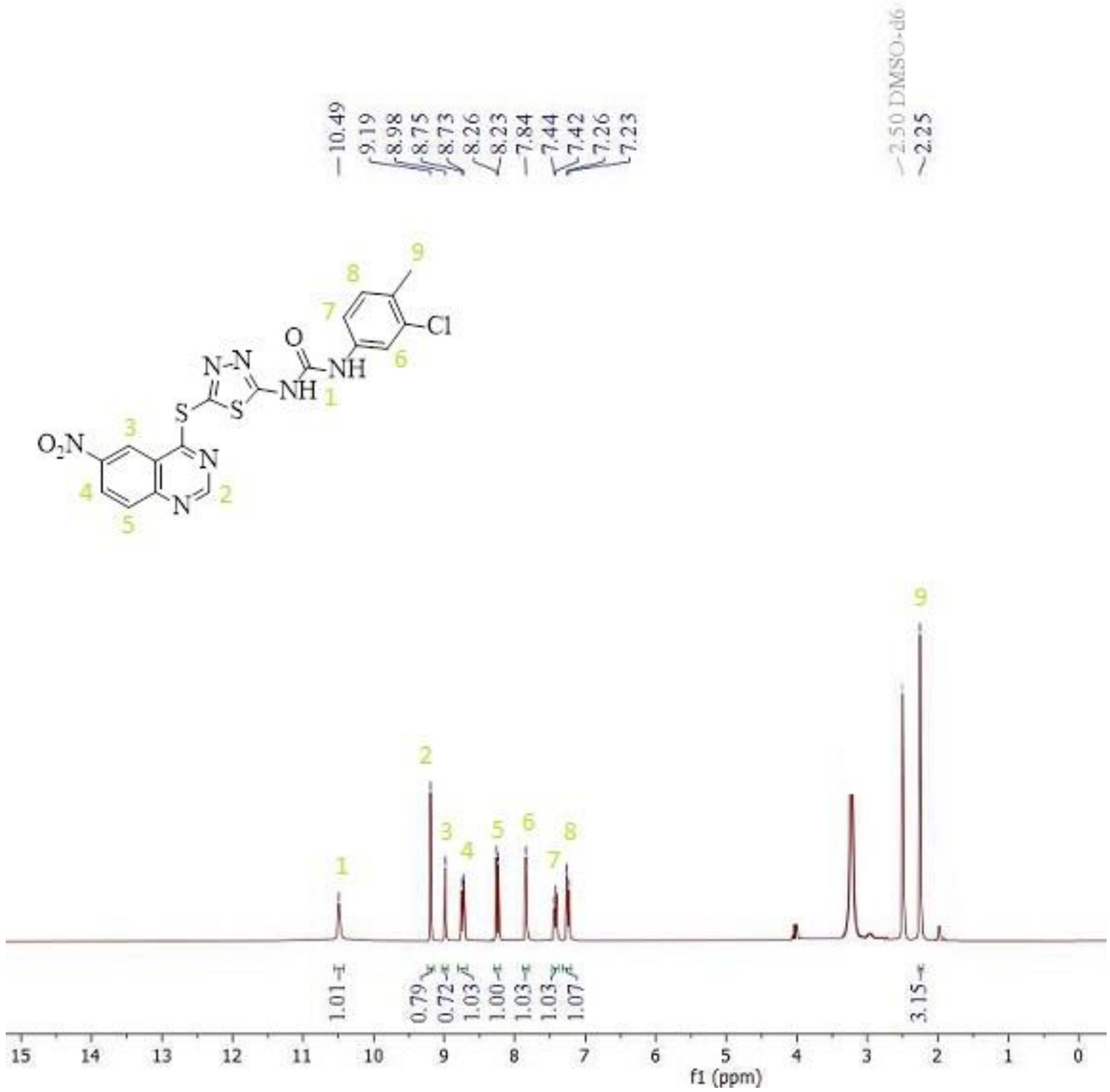

^{13}C NMR spectrum of 1-(3-Chloro-4-methylphenyl)-3-(5-((6-nitroquinazolin-4-yl)thio)-1,3,4-thiadiazol-2-yl)urea (8k)

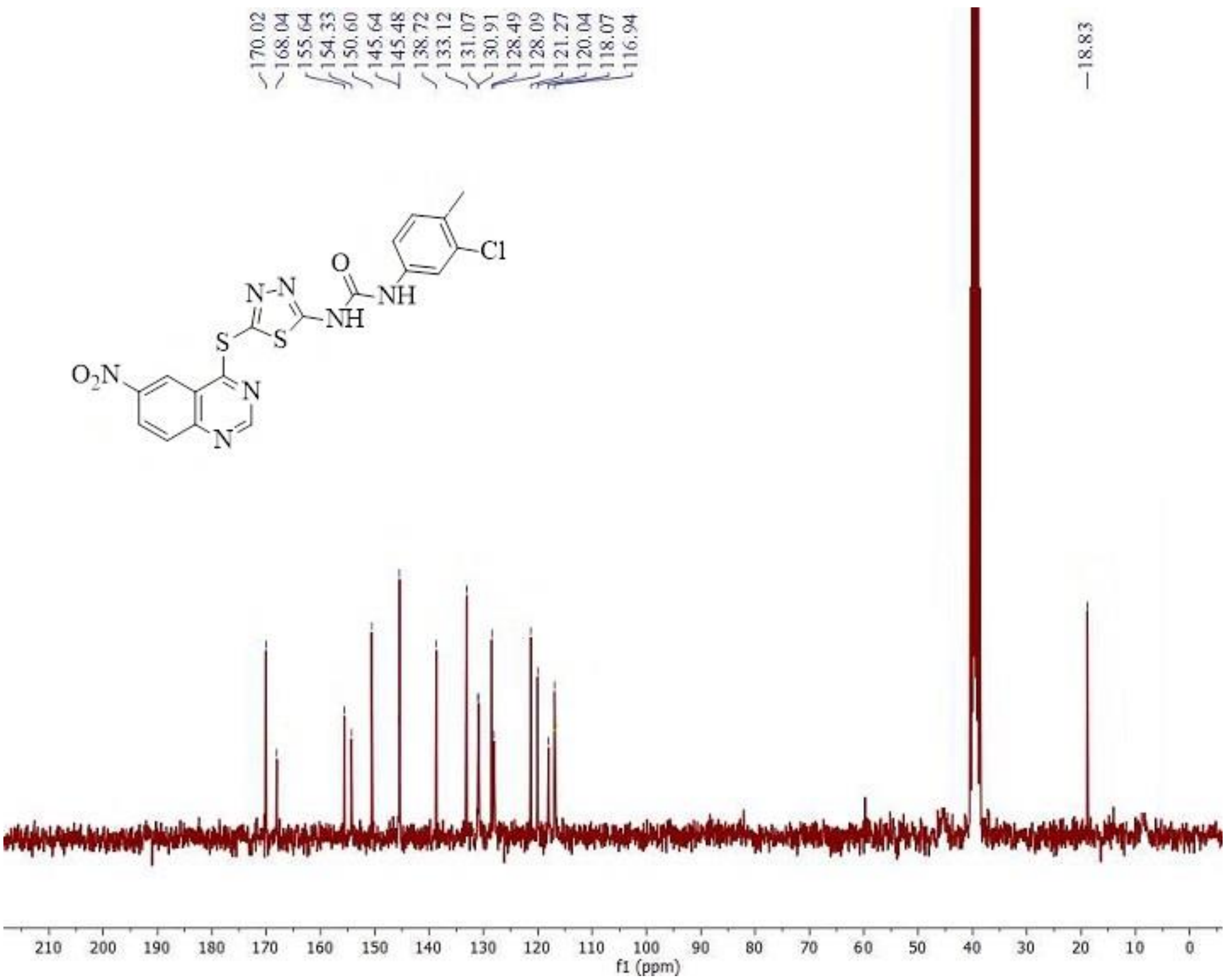

^{1}H NMR spectrum of 1-(4-chloro-3-(trifluoromethyl)phenyl)-3-(5-((6-nitroquinazolin-4-yl)thio)-1,3,4-thiadiazol-2-yl)urea (8l)

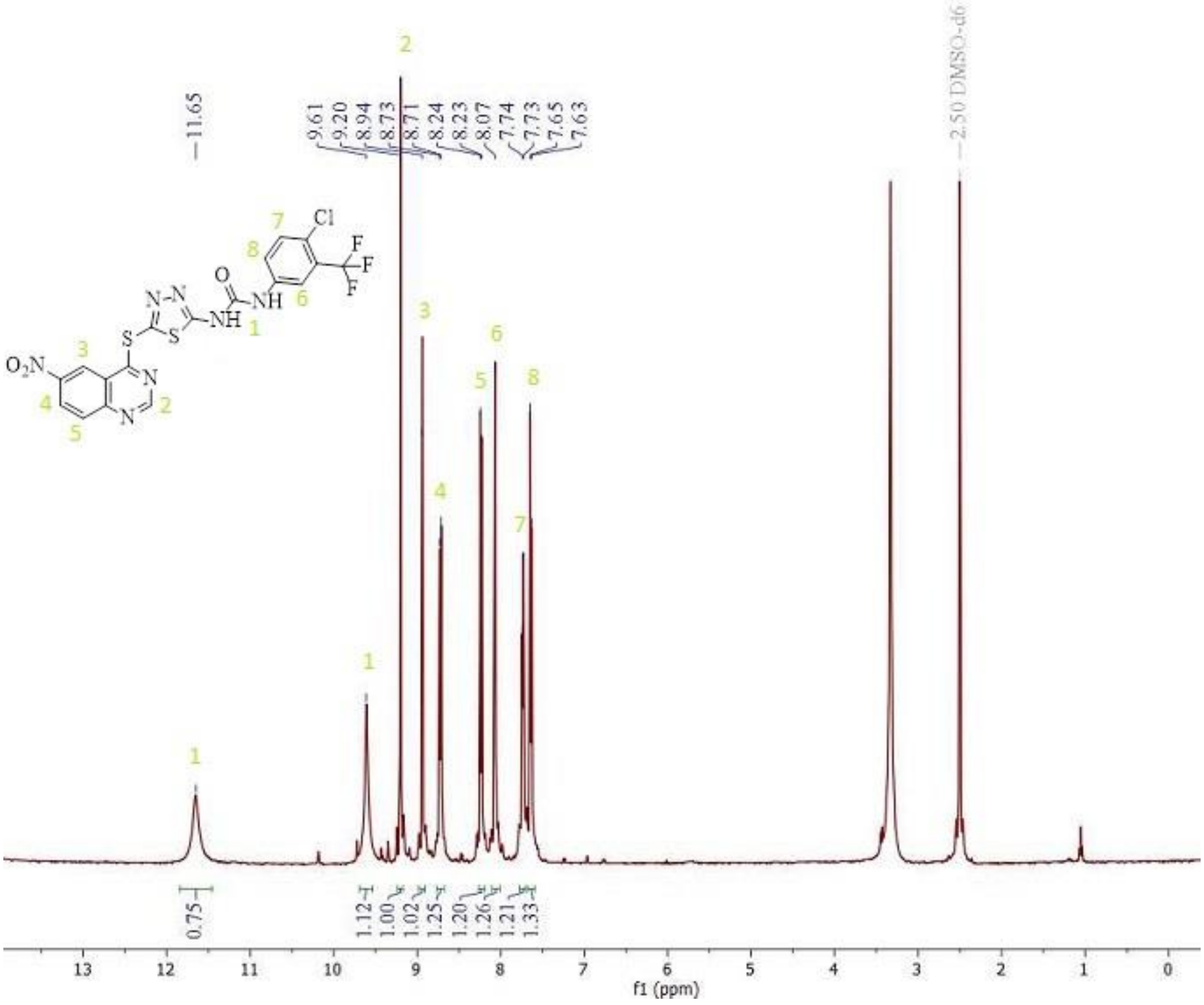

^{13}C NMR spectrum of 1-(4-chloro-3-(trifluoromethyl) phenyl)-3-(5-((6-nitroquinazolin-4-yl) thio)-1,3,4-thiadiazol-2-yl)urea (8l)

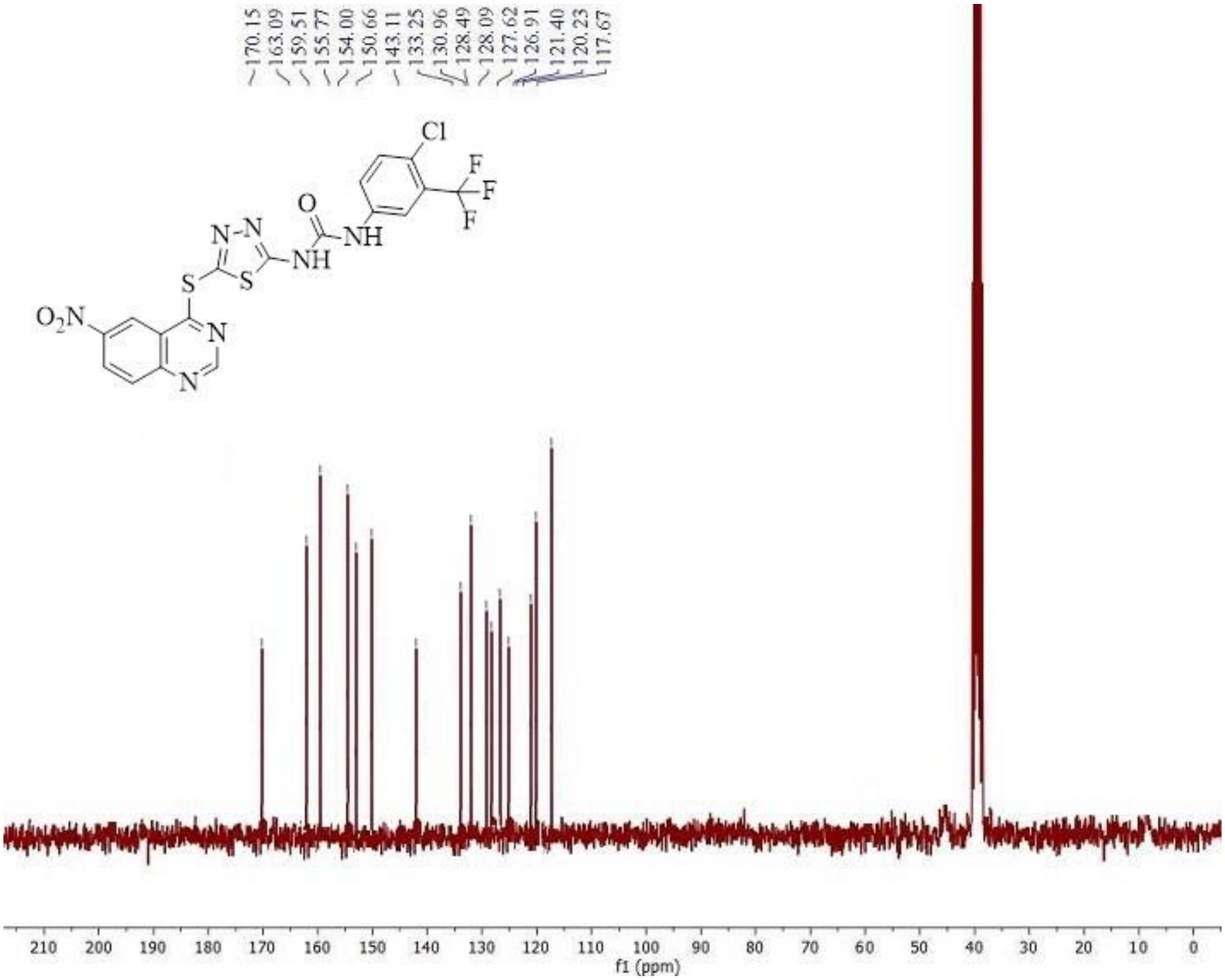

Supplement: Supplementary file 1 — Additional file 1. Additional file 1 of Novel quinazolines bearing 1,3,4-thiadiazole-aryl urea derivative as anticancer agents: Design, Synthesis, Molecular docking, DFT and Bioactivity evaluations [file 13065_2024_1119_MOESM1_ESM.pdf]
